# Compound Dynamics and Combinatorial Patterns of Amino Acid Repeats Encode a System of Evolutionary and Developmental Markers

**DOI:** 10.1093/gbe/evz216

**Published:** 2019-10-07

**Authors:** Ilaria Pelassa, Marica Cibelli, Veronica Villeri, Elena Lilliu, Serena Vaglietti, Federica Olocco, Mirella Ghirardi, Pier Giorgio Montarolo, Davide Corà, Ferdinando Fiumara

**Affiliations:** 1 Department of Neuroscience Rita Levi Montalcini, University of Torino, Italy; 2 National Institute of Neuroscience (INN), Torino, Italy; 3 Department of Translational Medicine, Piemonte Orientale University, Novara, Italy; 4 Center for Translational Research on Autoimmune and Allergic Disease (CAAD), Novara, Italy

**Keywords:** amino acid repeats, polyglutamine, polyalanine, HOX genes, evolution and development, homopolymeric

## Abstract

Homopolymeric amino acid repeats (AARs) like polyalanine (polyA) and polyglutamine (polyQ) in some developmental proteins (DPs) regulate certain aspects of organismal morphology and behavior, suggesting an evolutionary role for AARs as developmental “tuning knobs.” It is still unclear, however, whether these are occasional protein-specific phenomena or hints at the existence of a whole AAR-based regulatory system in DPs. Using novel approaches to trace their functional and evolutionary history, we find quantitative evidence supporting a generalized, combinatorial role of AARs in developmental processes with evolutionary implications. We observe nonrandom AAR distributions and combinations in HOX and other DPs, as well as in their interactomes, defining elements of a proteome-wide combinatorial functional code whereby different AARs and their combinations appear preferentially in proteins involved in the development of specific organs/systems. Such functional associations can be either static or display detectable evolutionary dynamics. These findings suggest that progressive changes in AAR occurrence/combination, by altering embryonic development, may have contributed to taxonomic divergence, leaving detectable traces in the evolutionary history of proteomes. Consistent with this hypothesis, we find that the evolutionary trajectories of the 20 AARs in eukaryotic proteomes are highly interrelated and their individual or compound dynamics can sharply mark taxonomic boundaries, or display clock-like trends, carrying overall a strong phylogenetic signal. These findings provide quantitative evidence and an interpretive framework outlining a combinatorial system of AARs whose compound dynamics mark at the same time DP functions and evolutionary transitions.

## Introduction

The evolutionary emergence of novel morphological and behavioral features in organisms constitutes a central biological problem (Gould 2002; Kirschner 2013), but the underlying genetic dynamics are only partially understood. Different types of mutations, including point mutations, transposon insertions, and replication slippage, in both coding and *cis*-regulatory parts of developmental genes, have been associated with morphological and behavioral evolution (Dover 1989; Pearson et al. 2005; Hoekstra and Coyne 2007; Carroll 2008; Lynch and Wagner 2008; Vinces et al. 2009).

In particular, replication slippage or unequal crossing-over in the coding part of developmental genes can induce the expansion or contraction of triplet repeats coding for homopolymeric amino acid repeats (AARs; Gemayel et al. 2010; Haerty and Golding 2010a, 2010b). Despite their abundance especially in developmental and nervous system proteins, often in pairwise or more complex combination (Green and Wang, 1994; Karlin and Burge 1996; Albà et al. 2007; Pelassa et al. 2014), their structures and possible functions are only partially understood.

AARs have often been held as intrinsically disordered spacers devoid of a specific structure/function and with a potential to misfold, causing disease, upon expansion (e.g., Wetzel 2012). However, early observations (e.g., Courey and Tjian 1988; Gerber et al. 1994) and a growing body of recent evidence are progressively increasing our understanding of the physiological roles of AARs. Several studies now show that AARs can form defined structures that mediate, or regulate, protein interactions, oligo-/poly-merization, localization and activity (e.g., Salichs et al. 2009; Fiumara et al. 2010; Gemayel et al. 2010, 2015; Schaefer et al. 2012; Pelassa and Fiumara 2015; Chavali et al. 2017; Mier et al. 2017; Lilliu et al. 2018; Escobedo et al. 2019). Moreover, AAR variation in certain proteins, such as RUNX2 and POU3F2, has been shown to regulate some aspects of morphology and behavior in metazoa (Treier et al. 1989; Galant and Carroll 2002; Fondon and Garner 2004; Anan et al. 2007; O’Malley and Banks 2008; Chew et al. 2012; Nasu et al. 2014; Hashizume et al. 2018). These findings suggested the hypothesis of an evolutionary role for AARs as regulatory “tuning knobs” modulating organismal morphology and behavior (Dover 1989; King et al. 1997; Kashi and King 2006; Haerty and Golding 2010b), also through epistatic interactions (Werner et al. 2006; Press et al. 2014; Press and Queitsch 2017).

However, it is unclear whether the modulatory effects on morpho-functional phenotypes are only occasional phenomena related to the scattered appearance of AARs in sparse developmental proteins (DPs) or, rather, hints of the existence of a whole system of functional AARs in DPs, their interactomes, and proteomes. If such a system exists, its contours are still essentially obscure and have to be traced at both the quantitative and qualitative levels.

In fact, despite the frequent occurrence of repeats of different amino acids in DPs (Karlin and Burge 1996), the specific functional meaning of each of them is still unclear. Notably, in this respect, the fact that polyQ expansion diseases are neurodegenerative diseases, and that polyA expansion diseases mostly cause skeletal and neurodevelopmental abnormalities (Almeida et al. 2013), suggests some degree of functional and regional specialization of proteins bearing different AARs, although this conclusion still remains purely conjectural.

Furthermore, while homopolymeric repeats of multiple amino acids, which can be structurally or functionally related, such as polyQ, polyA, and polyS, frequently co-occur in one same protein (Fondon and Garner 2004; Pelassa et al. 2014; Lilliu et al. 2018), the overall functional relevance of these AAR combinations is elusive.

Finally, the quantitative evolutionary dynamics of AARs and their combinations are not clearly understood, and their broad fluctuations across species/taxa (Faux 2012; Kumar et al. 2016) are largely enigmatic. In this regard, if AARs have been coopted as mediators of evolvability in metazoa (Dover 1989; King et al. 1997; Kirschner 2013), one may hypothesize that overall shifts in AAR occurrence and combination may have contributed to taxonomic divergence. In this case, some degree of regularity and phylogenetic signal should arguably be detectable in their quantitative evolutionary dynamics, rather than the apparently stochastic fluctuations that are reported in the literature. In addition, if AAR combinations are functionally relevant, one may hypothesize that the repeats of different amino acids may evolve as a whole system in an interrelated fashion, rather than as entirely independent sequences.

To address these issues, we use here novel approaches to trace the functional and evolutionary trajectories of the repeats of the 20 amino acids throughout phylogenesis and find quantitative and qualitative evidence supporting the existence of a generalized combinatorial system of AARs in developmental processes with evolutionary implications.

## Materials and Methods

### Datasets and Software

The amino acid sequences of 167 human DPs of interest, and their orthologs, were derived from Uniprot (www.uniprot.org; canonical isoforms; gene symbols: DLX1, DLX2, DLX3, DLX4, DLX5, DLX6, FOXA1, FOXA2, FOXA3, FOXB1, FOXB2, FOXC1, FOXC2, FOXD1, FOXD2, FOXD3, FOXD4, FOXE1, FOXE3, FOXF1, FOXF2, FOXG1, FOXH1, FOXI1, FOXI2, FOXI3, FOXJ1, FOXJ2, FOXJ3, FOXK1, FOXK2, FOXL1, FOXL2, FOXM1, FOXN1, FOXN2, FOXN3, FOXN4, FOXO1, FOXO3, FOXO4, FOXO6, FOXP1, FOXP2, FOXP3, FOXP4, FOXQ1, FOXR1, FOXR2, FOXS1, FOXD4L1, FOXD4L3, FOXD4L4, FOXD4L5, FOXD4L6, HOXA1, HOXA2, HOXA3, HOXA4, HOXA5, HOXA6, HOXA7, HOXA9, HOXA10, HOXA11, HOXA13, HOXB1, HOXB2, HOXB3, HOXB4, HOXB5, HOXB6, HOXB7, HOXB8, HOXB9, HOXB13, HOXC4, HOXC5, HOXC6, HOXC8, HOXC9, HOXC10, HOXC11, HOXC12, HOXC13, HOXD1, HOXD3, HOXD4, HOXD8, HOXD9, HOXD10, HOXD11, HOXD12, HOXD13, IRX1, IRX2, IRX3, IRX4, IRX5, IRX6, LHX1, LHX2, LHX3, LHX4, LHX5, LHX6, LHX8, LHX9, NKX1-1, NKX1-2, NKX2-1, NKX2-2, NKX2-3, NKX2-4, NKX2-5, NKX2-6, NKX2-8, NKX3-1, NKX3-2, NKX6-1, NKX6-2, NKX6-3, PAX1, PAX2, PAX3, PAX4, PAX5, PAX6, PAX7, PAX8, PAX9, POU1F1, POU2F1, POU2F2, POU2F3, POU3F1, POU3F2, POU3F3, POU3F4, POU4F1, POU4F2, POU4F3, POU5F1, POU5F1B, POU5F2, POU6F1, POU6F2, SOX1, SOX2, SOX3, SOX4, SOX5, SOX6, SOX7, SOX8, SOX9, SOX10, SOX11, SOX12, SOX13, SOX14, SOX15, SOX17, SOX18, SOX21, SOX30, SRY). Reference proteomes were retrieved from Uniprot without isoforms for the following 55 species: *H. sapiens* (Hom sap), *Pan troglodytes* (Pan tro), *Pongo abelii* (Pon abe), *Callithrix jacchus* (Cal jac), *Otolemur garnetti* (Oto gar), *Mus musculus* (Mus mus), *Rattus norvegicus* (Rat nor), *Heterocephalus glaber* (Het gla), *Ailuropoda melanoleuca* (Ail mel), *Felis catus* (Fel cat), *Bos taurus* (Bos tau), *Ovis aries* (Ovi ari), *Sus scrofa* (Sus scr), *Monodelphis domestica* (Mon dom), *Sarcophilus harrisii* (Sar har), *Ficedula albicollis* (Fic alb), *Taeniopygia guttata* (Tae gut), *Gallus gallus* (Gal gal), *Meleagris gallopavo* (Mel gal), *Anas platyrhynchos* (Ana pla), *Anolis carolinensis* (Ano car), *Ophiophagus hannah* (Oph han), *Astyanax mexicanus* (Ast mex), *Danio rerio* (Dan rer), *Oryzias latipes* (Ory lat), *Xiphophorus maculatus* (Xip mac), *Oreochromis niloticus* (Ore nil), *Gasterosteus aculeatus* (Gas acu), *Takifugu rubripes* (Tak rub), *Tetraodon nigroviridis* (Tet nig), *Lepisosteus oculatus* (Lep ocu), *Apis mellifera* (Api mel), *Camponotus floridanus* (Cam flo), *Acromyrmex echinatior* (Acr ech), *Atta cephalotes* (Att cep), *Solenopsis invicta* (Sol inv), *Anopheles gambiae* (Ano gam), *Anopheles darlingi* (Ano dar), *Aedes aegypti* (Aed aeg), *Drosophila pseudoobscura* (Dro pse), *Drosophila persimilis* (Dro per), *Drosophila mojavensis* (Dro moj), *Drosophila virilis* (Dro vir), *Drosophila grimshawi* (Dro gri), *Drosophila melanogaster* (Dro mel), *Drosophila sechellia* (Dro sec), *Drosophila simulans* (Dro sim), *Caenorhabditis remanei* (Cae rem), *Caenorhabditis brenneri* (Cae bre), *Caenorhabditis briggsae* (Cae bri), *Caenorhabditis elegans* (Cae ele), *Caenorhabditis japonica* (Cae jap), *Komagataella pastoris* (Kom pas), *Saccharomyces cerevisiae* (Sac cer), and *Schizosaccharomyces pombe* (Sch pom). Protein sequences were then analyzed using ad hoc Perl scripts (www.perl.org). Standard eukaryotic phylogenies and phylogenetic distances were derived from www.timetree.org (Hedges et al. 2006), using the median divergence times. For the primate/ecdysozoa divergence time, we used the value of 626.5 mya from a recent study reported in TimeTree (dos Reis et al. 2015), given the broad confidence interval of the estimate.

### Analysis of AAR Occurrence and co-Occurrence

For each proteome, we calculated the number of proteins containing a repeat of at least 4 units of each amino acid (X_4_, where X is one of the 20 amino acids) and pairwise AAR combinations (X_4_+Z_4_, where X and Z are two different amino acids). Percent values [%X_4_ and %(X_4_+Z_4_)] were calculated by normalizing to the number of proteins in the proteome.

The statistical significance of the pairwise co-occurrence of AARs in each proteome was calculated by using the χ^2^ test with a Benjamini-Hochberg correction (FDR = 0.05). We also calculated all the possible pairwise repeats ratios (RR parameters) between %X_4_s [i.e., RR(X_4_/Z_4_) = (%X_4_/%Z_4_) where X and Z are two amino acids] as an index of the relative occurrence of two AARs in a proteome (Pelassa et al. 2014). Moreover, as indexes of AAR cooccurrence (OV parameters), we calculated the relative proportion of proteins containing a given repeat that also contain another repeat [%(X_4_+Z_4_)/%X_4_] and then normalizing this value to the size (%X_4_) of the group containing the given repeat [i.e. OV(X_4_+Z_4_) = %(X_4_+Z_4_)/(%X_4_)^2^; Pelassa et al. 2014].

We thus calculated 400 RR and 400 OV parameters for each proteome in 20 × 20 matrices. In these matrices, particular cases were observed on the diagonals that is, the 20 RR(X_4_/X_4_) parameters, which all equal 1 and do not carry any signal for cluster analyses, and by the 20 OV(X_4_+X_4_) parameters whose value corresponds to 1/X_4_. These latter cases were also included in the cluster analyses as they carry some phylogenetic signal related to X_4_s. Cluster analyses were thus performed using all the 800 parameters (or a subset of 128 of them derived from the polyA/E/G/H/P/Q/R/S, see Results section). RRs and OVs in which a value at the denominator of a division operation was equal to 0 were assigned a null value of 0. The same approach was used to calculate the same set of parameters for sets of 20 random tetrapeptides (RND_4_), generated either by randomly reshuffling the 20 homopetides or by replacing the second, third, and fourth residue of each homopeptide with randomly selected amino acids (fig. 5*E* andsupplementary fig. S8*B*, Supplementary Material online). Amino acid usage in each proteome was calculated using a Perl script counting the proteome-wide occurrences of each amino acid.

### Analysis of AAR Occurrence in Vertebrate DP Orthologs

Ortholog sequences of the 167 human DPs were downloaded from Ensembl and the percentage of the orthologs of each DP containing the AARs of interest was quantified in each taxon of interest. To maximize sensitivity, even partial sequences were included, although some AARs may thus be apparently missing in some species/taxa owing to sequence incompleteness.

### Interactome Analysis

The interactomes (physical interactions) of 167 DPs and 167 control MPs were derived from BioGrid (Chatr-Aryamontri et al. 2015). MPs were chosen randomly using an ad hoc script among lists of proteins associated with the GO terms (GO0005975 and GO0006629) derived from Panther (Mi et al. 2013). The binary PPIs of interest were represented in graphs using Cytoscape (Shannon et al. 2003). Proteins containing AARs of interest were represented as red nodes, and the interactions between red nodes as red edges. Interactomes were represented as two concentric circles (fig. 2*A*), with the inner circle formed by either DPs or MP nodes, and the outer circle formed by their respective interactors.

### Cluster Analysis and Principal Component Analysis

%X_4_, RR(X_4_/Z_4_), and OV(X_4_+Z_4_) parameters, or their mean values in taxa of interest, were used to define clusters of covarying phylogenetic profiles of AAR occurrence/cooccurrence across taxa, and clusters of taxa/species based on AAR occurrence/cooccurrence profiles, using Cluster 3.0 (de Hoon et al. 2004), helding the AAR-related parameters as “genes” and taxa/species as “arrays.” Data were adjusted by normalizing “genes” and “arrays,” centering “genes” (mean), and clustered hierarchically using “Spearman rank correlation” and “average linkage.” The same approach was used in control analyses with random tetrapeptides. To identify fundamental components to the phylogenetic AAR variation, a principal component analysis (PCA) was performed using SPSS 21 with varimax rotation and 25 maximum iterations per convergence.

### Analysis of the Relation between AARs and DP Functions in Human Proteins

We investigated the preferential associations of AARs with specific DP functions by determining whether protein sets associated with a specific developmental GO term, or with a cluster of semantically related GO terms, contained a significant overrepresentation of AAR-containing proteins, as assessed by means of χ^2^ tests on 2 × 2 contingency tables followed by a Benjamini-Hochberg correction for multiple testing (FDR = 0.05; Benjamini and Hochberg 1995). The Uniprot identifiers of human protein sets associated with GO terms containing the string “development,” were downloaded from AmiGO (Carbon et al. 2009). Because certain GO terms are associated with a small number of proteins, thus limiting the statistical power of the analysis, we obtained larger sets of functionally related proteins by pooling sets of proteins associated with semantically related GO terms. Thus, 655 developmental GO terms were manually grouped into 19 large clusters related to general or system-specific developmental processes and 131 smaller subclusters in relation to organs/parts of larger systems (supplementary table S2, Supplementary Material online). The protein identifiers associated with the GO terms in one (sub)cluster were pooled and analyzed statistically as described. The results of these analyses were plotted as network graphs generated using Cytoscape (Shannon et al. 2003) or clustered using Cluster3.0 and TreeView (Saldanha 2004), emploing χ^2^ values as indicators of the strength of each AAR-(sub)cluster association, as described in the Results section.

### Analysis of the Evolutionary Dynamics of the AAR Functional Associations

To analyze dynamic phylogenetic changes of the preferential AARs/DP function associations, we focused on 5 representative GO terms (GO: 0009790/embryo development, GO: 0007389/pattern specification process, GO: 0001501/skeletal system development, GO: 0007399/nervous system development, GO: 0007399/heart development) and two control terms (GO: 0005975/carbohydrate metabolic process; GO: 0006629/lipid metabolic process). The Uniprot identifiers of proteins associated with these GO terms in species of interest were downloaded from Panther (Mi et al. 2013). The fold enrichment, with respect to the whole proteome, of proteins associated with a specific GO term among proteins bearing a certain AAR was calculated and its statistical significance was assessed by means of χ^2^ tests on 2 × 2 contingency tables.

### Data Analysis, Graphs, and Statistics

Data were processed and analyzed statistically using Excel (Microsoft), Prism (GraphPad), and SPSS 21 (IBM) software. Appropriate statistical tests were performed as indicated in the Results section and *P* < 0.05 was considered as statistically significant in all instances. The Benjamini-Hochberg procedure (Benjamini and Hochberg 1995), where appropriate, was also used to control for the FDR. The FDR rate was set to 0.05 in all instances.

Graphs and figures were generated using Excel (Microsoft), Igor Pro 6.1 (WaveMetrics Inc.), Cytoscape, Java TreeView (Saldanha 2004), MyDomains (Sigrist et al. 2013), Photoshop Elements 11 (Adobe) or InkScape software. Protein alignments were produced using Clustal Omega software (Sievers et al. 2011). Unscaled unrooted phylogenetic trees showing the relationships between species/taxa, as derived from cluster analysis dendrograms, were drawn as graphs, where nodes represent species/taxa and edges represent their mutual releationships, using Cytoscape (organic layout). Silhouettes of animal species in figure 4D were obtained from Phylopic.org (credits: *Gorilla gorilla* by T. Michael Keesey (after Colin M. L. Burnett), *Mus musculus* by Daniel Jaron, *Bos primigenius* taurus by Steven Traver, Sauropsida by Nobu Tamura (vectorized by T. Michael Keesey; https://creativecommons.org/licenses/by-sa/3.0/), and Eupercaria by Lily Hughes.

## Results

### Differential Overrepresentation of AARs and Their Combinations in HOX and Other Major DP Families

**Fig. 1.**
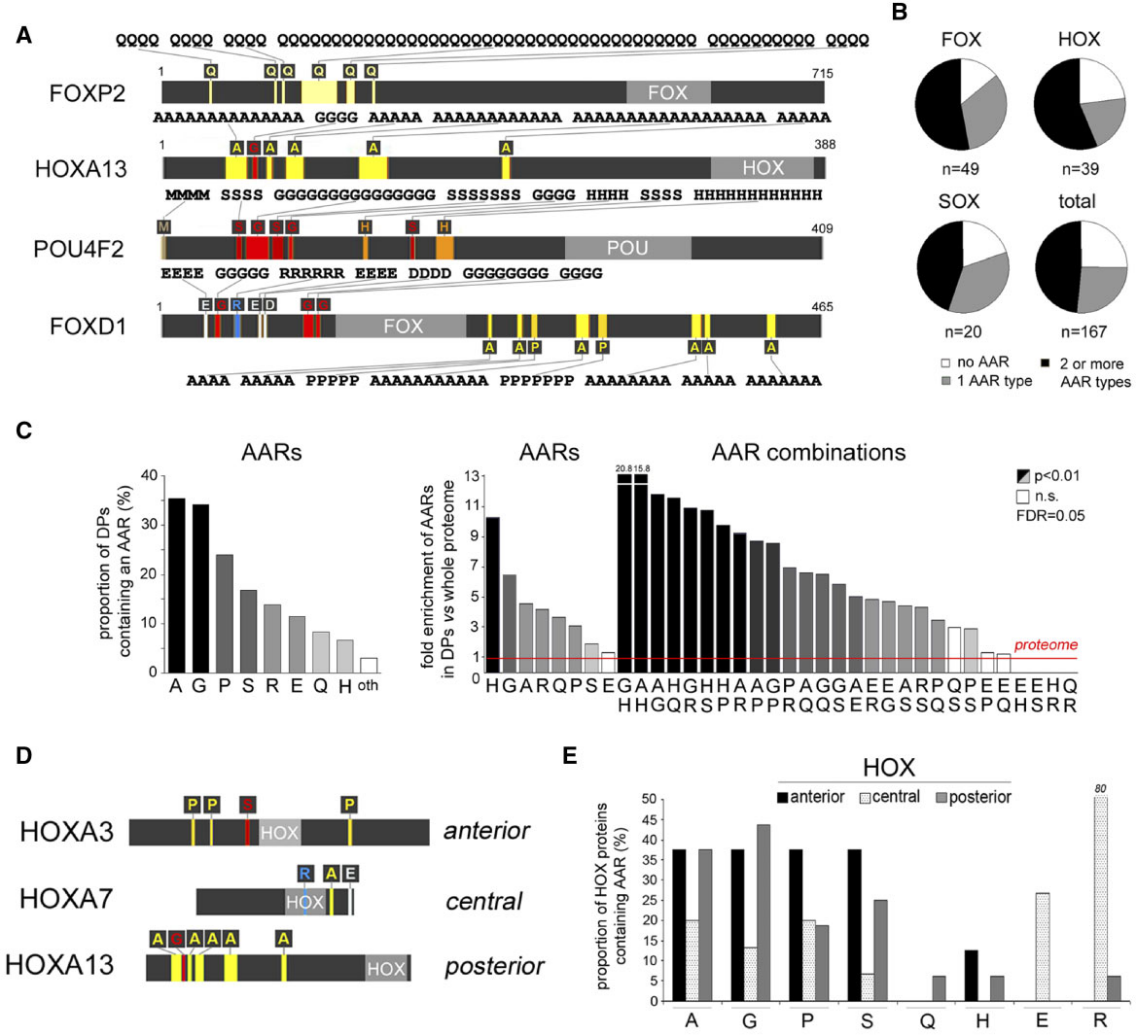
—Differential distribution of AARs and their pairwise combinations in HOX and other major DP families. (*A*) Schematic representation (gray bars) of selected human DPs. AARs are represented by colored stripes and one-letter amino acid symbols above/below each bar. AAR sequences are reported above/below the bars. Forkhead (FOX), homeobox (HOX), and POU domains are in light gray. The numbers on the right indicate the total number of residues in each protein. (*B*) Pie charts representing the proportion of proteins with no AAR, AARs of one amino acid only, or two/more, in the HOX, FOX, SOX families and in the whole set of 167 DPs. (*C*) The left histogram displays the percentage of proteins in the 167 DP set that contains the indicated AARs. The right histogram displays the fold enrichment of the indicated AARs, or their pairwise combinations, in the DP set in comparison with the whole proteome (red line). Abbreviations: *p*, *P* value; n.s., nonsignificant; FDR, Benjamini-Hochberg false discovery rate. (*D*) Schematic representation as in (*A*) of selected HOX proteins of the anterior, central, and posterior classes. The length of each bar is proportional to the protein length. (*E*) Histogram displaying the percentage of proteins in the anterior, central, and posterior classes of HOX proteins containing the indicated AARs.

Although the frequent occurrence of AARs in DPs and neuronal proteins was observed early on in AAR studies (Karlin and Burge 1996), a precise quantitative understanding of AARs, and especially of their combinations, in DPs is still lacking. Because the known AARs with regulatory roles in development are in transcription factors (TFs) belonging to the HOX, POU, and other families (Treier et al. 1989; Galant and Carroll 2002; Fondon and Garner 2004; Anan et al. 2007; O’Malley and Banks 2008; Chew et al. 2012; Nasu et al. 2014; Hashizume et al. 2018), we focused our investigation on a set of TFs with known developmental roles and belonging to relatively large and functionally characterized families. We thus started our analysis by focusing on nine major human DP families of TFs, i.e. HOX, FOX, SOX, PAX, DLX, POU, IRX, LHX, and NKX, with established, major developmental roles (fig. 1 and supplementary figs. S1 and S2, Supplementary Material online), for a total of 167 DPs. We determined whether each one of these 167 DPs contains a repeat of at least four residues of a given amino acid (X_4_, where X is for any amino acid). We performed this count for all of the 20 amino acids (fig. 1*A* and *B* and supplementary fig. S1*A* and *B*, Supplementary Material online). This AAR length threshold allows one to capture repeats at different stages of their life cycle (Buschiazzo and Gemmell 2006; Pelassa et al. 2014), including regions of cryptic simplicity with fragmented AARs.

We found that while some proteins contain a single type of AAR, often in multiple stretches (e.g., polyQ in FOXP2), other contain pairwise or higher order combinations (e.g., HOXA13, POU4F2, FOXD1; fig. 1*A* andsupplementary fig. S1*A*, Supplementary Material online). Overall, the majority of these DPs contain at least one type of AAR, and about half of them contain combinations of two or more (fig. 1*B* andsupplementary fig. S1*B*, Supplementary Material online). In DP families like HOX, FOX, and SOX, AARs are present in more than two thirds of the proteins. AARs and their combinations occur with variable frequencies in the different DP families. Overall, AARs of alanine (A), glutamate (E), glycine (G), histidine (H), proline (P), glutamine (Q), arginine (R), and serine (S) occur more frequently in these proteins (fig. 1*C*).

Seven of the eight more abundant repeats (A, G, H, P, Q, R, S; fig. 1*C*) are significantly overrepresented in the DP set in comparison with the whole proteome (from 1.8 to 10.2 times, *P* < 0.01 in all instances, χ^2^ test with Yates correction, Benjamini-Hochberg false discovery rate [FDR] = 0.05). Notably, a considerable number of pairwise AAR combinations are also highly overrepresented, from 2.9 to 20.8 times, with respect to the whole proteome (*P* < 0.01, fig. 1*C*).

Among the DP families, the HOX, FOX, SOX, and POU proteins display higher degrees of overall AAR occurrence (supplementary fig. S1*C*, Supplementary Material online). Interestingly, an overall statistical analysis of the occurrence in these protein families of the eight more represented AARs (polyA/E/G/H/P/Q/R/S) revealed that polyA, polyE, polyG, and polyR are differentially distributed across the nine DP families (*P* < 0.05 in all instances, Fisher exact [FE] test). For the other four AARs, the differences were not statistically significant, likely due to the relatively low number of proteins in each group, although quite large absolute variations were evident in the percentage of these AARs across DP families.

We then focused on the HOX DP family, that can be further divided into three functionally distinct subfamilies involved in the development of anterior, central, and posterior body segments (Duboule 1994) (fig 1*D* and supplementary fig. S2, Supplementary Material online). Notably, the distribution of AARs differs considerably among the three human HOX subfamilies. Indeed, while charged AARs are found mostly in central HOX proteins (*P* < 0.001, Fisher’s exact test), combinations of two or more small, polar, and cyclic AARs are significantly more frequent in the antero-posterior group (*P* < 0.02, FE test; fig. 1*E* and supplementary figs. S1*D* and S2, Supplementary Material online).

Taken together, these findings indicate that some AARs of polar (Q, S), charged (E, H, R), small (A, G), and cyclic (P) residues are differentially overrepresented in human DP families and subfamilies, in a combinatorial manner, with a high prevalence of pairwise and higher order AAR combinations.

### Parallel Overrepresentation of AARs Mediating Protein–Protein Interactions in the Interactomes of DPs

AARs can mediate homotypic interactions with other AARs of the same kind in other proteins, and heterotypic interactions with conventional protein–protein interaction (PPI) domains (e.g. Pelassa and Fiumara 2015). Thus, it is conceivable that the observed overrepresentation of AARs, and their combinations, in DPs may underlie the formation of interaction networks with their protein partners.

**Fig. 2.**
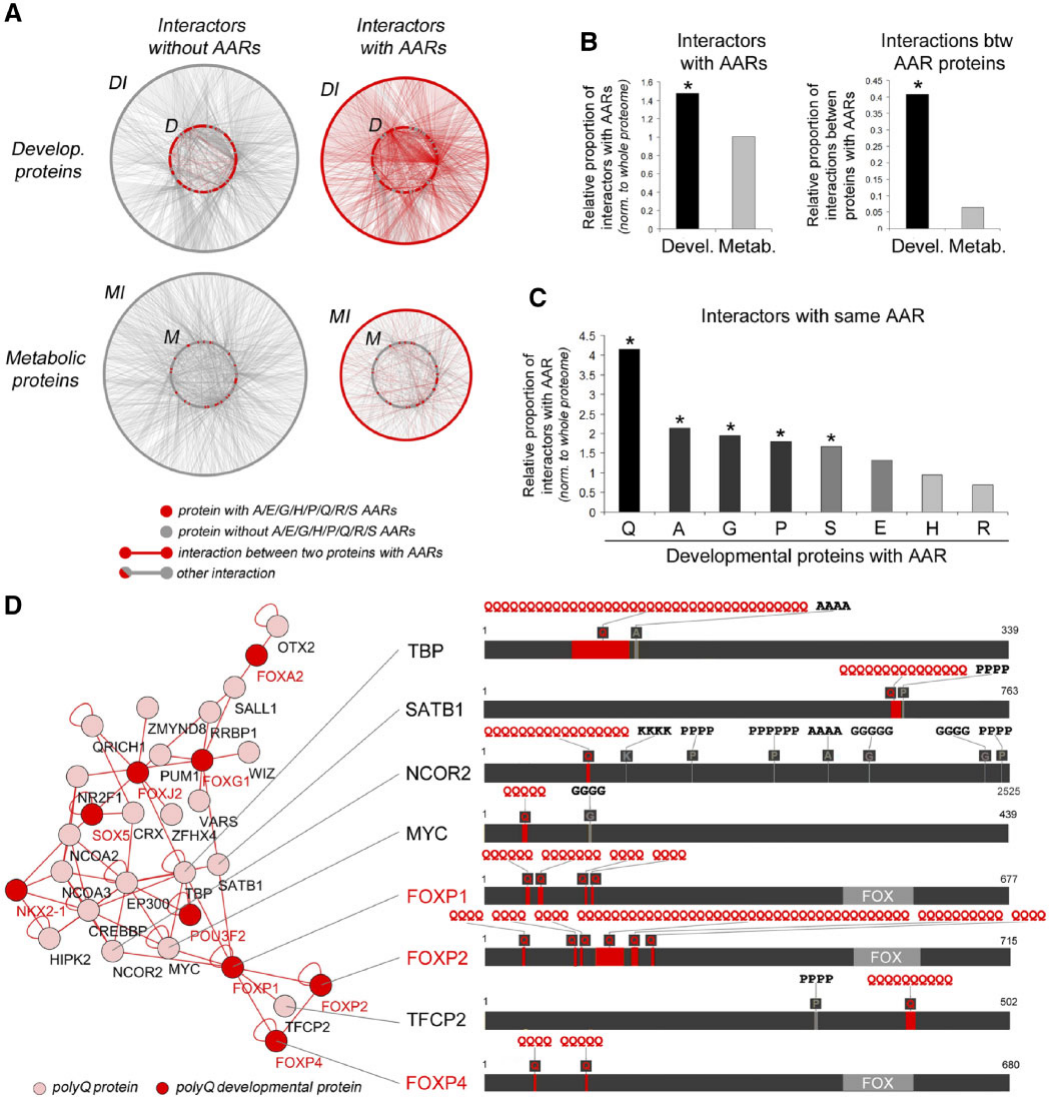
—Overrepresentation of PPI-mediating AARs in the interactome of DPs. (*A*) Compact graph representation of the interactomes of 167 DPs and 167 control MPs randomly chosen among those involved in carbohydrate and lipid metabolism, in which individual proteins are represented as adjacent dots forming circles. Each protein dot is colored in red, if the protein contains an AAR, or in gray if not. DPs (*D*) form the inner circle in the upper graphs, MPs (*M*) form the inner circles in the lower *graphs*. Their interactors (DI and MI, respectively) are represented in the external circles. Gray edges connecting pairs of proteins represent PPIs, which are in red if connecting two proteins with AARs. (*B*) The left histogram shows the relative proportion of interactors of proteins bearing the indicated AARs that contain that same AAR (e.g., proportion of polyQ proteins among the interactors of polyQ-containing DPs). Values are normalized to the proportion of proteins containing the same AAR in the whole proteome. The right histogram shows the relative proportion of interactions between proteins that contain AARs. Asterisks mark significant overrepresentations. (*C*) Histogram representing the relative proportion of A/E/G/H/P/Q/R/S AAR-containing proteins in the interactomes of DPs and MPs shown in *A*. Values are normalized to the proportion of proteins containing the same AARs in the whole proteome. Asterisks mark significant overrepresentations. (*D*) Graph representation (left panel) of an interactome formed by polyQ-containing DPs and their polyQ interactors. The right panel highlights a subnetwork of FOXP1 interactors, representing their AARs as in Figure 1*A*.

In search of evidence supporting this hypothesis, we analyzed whether the known interactomes of human DPs are in fact enriched with proteins bearing the same types of AARs (fig. 2 and supplementary fig. S3, Supplementary Material online). We extracted from the human interactome in Biogrid (fig. 2*A*) the physical interactions between the 167 DPs (*D*) and their direct interactors (DI), and those between a control set of 167 metabolic proteins (MPs, *M*), randomly chosen among those involved in carbohydrate/lipid metabolism (see Materials and Methods section), and their interactors (MI). The average number of interactions formed by each protein in the two groups was comparable (21.12 ± 3.32 vs 17.62 ± 2.18 for the DP and MP groups, respectively, *P* = 0.37 Student’s *t*-test). We found that, unlike MPs, DPs have a considerably higher proportion of interactors containing A, E, G, H, P, Q, R, S repeats (fig. 2*A* and *B*) than expected by chance based on the proteome-wide occurrence of the same AARs (χ^2^= 161.62, *P* < 0.0001, χ^2^ test, for DPs; *P* = 0.97 for MPs, fig. 2*B*, left panel). Moreover, also the proportion of interactions between AAR-bearing proteins is much higher in the DP than in the MP interactome (*P* < 0.001, χ^2^ test; fig. 2*B*, right panel).

Notably, DPs containing either Q, A, P, G, or S repeats, but not charged E, H, or R repeats, have a proportion of interactors with the same AAR higher than expected based on the overall proteome-wide occurrence of the AAR (*P* < 0.001, χ^2^ test; fig. 2*C* and supplementary fig. S3*A*, Supplementary Material online). In fact, DPs containing either polyQ (fig. 2*D*), polyA (supplementary fig. S3*B*, Supplementary Material online), polyG, polyP, or polyS repeats (supplementary fig. S3*C*, Supplementary Material online) are part of complex interaction networks with other proteins bearing the same type of AAR.

These findings show a parallel enrichment of PPI-mediating AARs (A, G, P, Q, S; Pelassa and Fiumara 2015) in human DPs and their interactors, consistent with the notion that they may be relevant to the establishment of functional PPI networks. Conversely, charged AARs may be functional in modulating additional electrostatic DP interactions with charged targets such as DNA and histones or other AARs bearing an opposite charge (Pelassa and Fiumara 2015; see Discussion section).

### AARs and Their Combinations as Markers of DP Functions

**Fig. 3.**
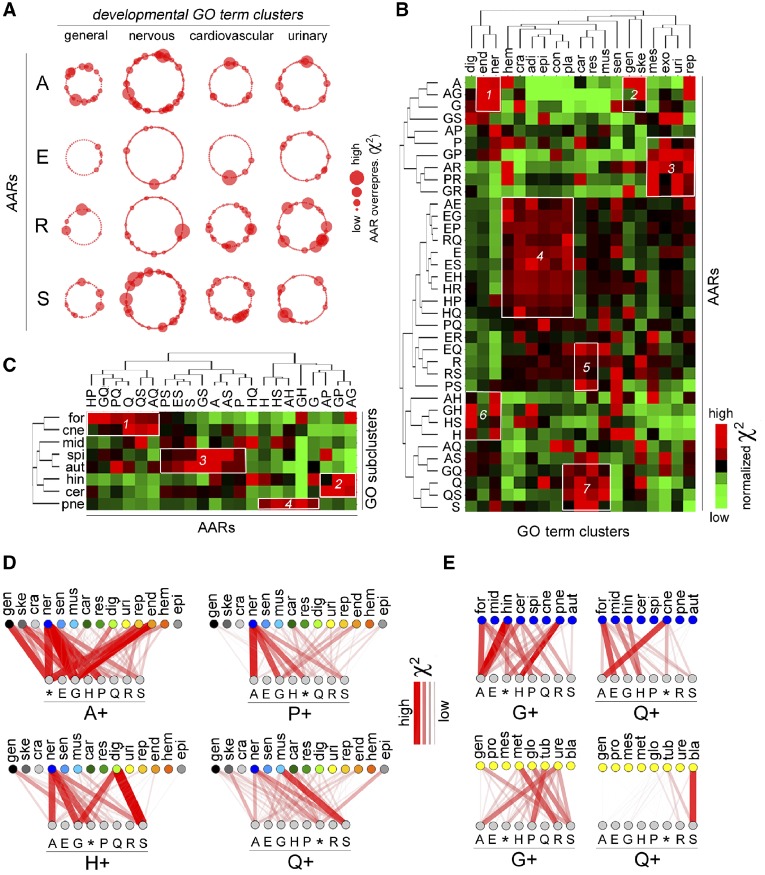
—Preferential combinatorial associations between AARs and DP functions. (*A*) Schematic representation of the overrepresentation of AAR-bearing proteins (A, E, R, and S AARs) in protein sets associated with developmental GO terms. Individual GO terms are represented by dots, grouped in four clusters (rings) associated with “general” or system-specific (“nervous,” “cardiovascular,” and “urinary”) developmental processes. The diameter of each dot is proportional to the AAR overrepresentation (χ^2^ value) in the set of proteins associated with the GO term. (*B*) Heat map of the association between AARs (rows) and proteins belonging to the 19 clusters (columns) involved in general (*gen*) or system/organ-specific developmental processes (e.g., nervous system, ner. Other abbreviations: adi, adipose tissue; car, cardiovascular system; con, connective tissue; cra, cranium; dig, digestive system; end, endocrine system; epi, epithelia/epidermis/adnexa; exo, exocrine glands; hem, hematopoietic/lymphatic systems; mes, mesenchyme/stem cells; mus, muscle; pla, placenta; rep, reproductive system; res, respiratory system; sen, sensory organs/systems; ske, skeleton; uri, urinary system). Each square represents normalized mean-centered χ^2^ value of each AAR/cluster association on a color scale where bright red represents highly significant associations and bright green non-significant associations. Black squares and those with darker shades of red and green represent intermediate levels of association. Note that black, in this normalized mean-centered heat map, represents intermediate levels of relative χ^2^ value in each cluster and not a threshold for statistical significance. For absolute χ^2^ values and statistical significance see Table S3. (*C*) Heat map of the association between AARs (columns) and proteins belonging to subclusters (rows) associated with the development of specific parts of the nervous system (e.g., forebrain, for). Each square represents the subcluster-normalized χ^2^ value of each AAR/subcluster association as in (*B*). Other abbreviations: aut, autonomous nervous system; cer, cerebellum; cne, cranial nerves; for, forebrain; hin, hindbrain; mid, midbrain; pne, peripheral nervous system; spi, spinal cord. (*D* and *E*) Graphs representing the strength of the statistical association between AAR combinations and developmental GO term clusters (*D*) or subclusters (*E*). The thickness of the lines connecting the nodes is proportional to the χ^2^ value of each association. The upper rows indicate GO term (sub)clusters. The lower rows indicate the combinations of a given AAR, indicated below the horizontal line (e.g., A+), with the other AARs indicated above. The asterisk indicates the overall set of proteins with a given AAR (e.g., A+* indicates polyA proteins overall, irrespective of their combination with other AARs). Cluster and subcluster abbreviations as in (*B* and *C*). Other abbreviations: bla, bladder; cel, cellular processes; clo, cloaca; gen, urinary system in general; glo, glomeruli; mes, mesonephros; met, metanephros; pro, pronephros; tub, tubules; ure, ureter.

If AARs in DPs and their interactomes specify PPI networks, it is then possible that specific AARs and their combinations may mark interacting, functionally related, protein sets with roles in the development of specific anatomical systems, rather than DPs in general. To test this hypothesis, we performed a comparative analysis of the relative enrichment of DP-related AARs (poly-A/-E/-G/-H/-P/-Q/-R/-S) of at least four residues in human protein sets associated with the development of anatomical (sub)systems and their parts (fig. 3, supplementary fig. S4, Supplementary Material online, and supplementary tables S1–S3, Supplementary Material online).

We obtained from the AmiGO database (Carbon et al. 2009) sets of human proteins associated with developmental “biological process” gene ontology (GO) terms that were semantically grouped in 19 clusters based on their relation to general (“general” cluster) or system-specific (e.g., “nervous system” cluster) developmental processes. These clusters were further subdivided in 131 subclusters associated with the development of specific parts/organs of each system (e.g., “forebrain” subcluster within the “nervous system” cluster; supplementary table S2, Supplementary Material online).

We then performed χ^2^ tests for the protein sets within each (sub)cluster in order to detect enrichments of AAR-containing proteins in comparison with the whole proteome.

In an initial overall screening, we represented the sets of proteins associated with each individual GO term as dots whose size is proportional to the χ^2^ value that is, to the degree of overrepresentation of proteins bearing a given AAR in them. These dots were grouped in rings representing each GO term cluster. This analysis highlighted how certain AARs are enriched in numerous protein sets involved in the development of some anatomical systems but not of others (fig. 3*A*). For instance, polyA is frequently overrepresented in proteins sets involved in general and nervous system-related developmental processes, rather than in cardiovascular or urinary system-related processes. The opposite is true for polyR, while polyE repeats are scarcely represented in all of these protein sets. These initial findings prompted us to perform a systematic analysis of the preferential enrichments of AARs in developmental processes associated with each GO term (sub)cluster using χ^2^ tests with a Benjamini-Hochberg correction (FDR = 0.05). This analysis revealed complex, combinatorial patterns of AAR enrichment in protein sets associated with the development of specific anatomical systems and their parts/organs (fig. 3*B*–*E*, supplementary fig. S4, Supplementary Material online, and supplementary table S3, Supplementary Material online).

To gain a synoptic view of these differential enrichments, we performed a cluster analysis of the AARs versus the 19 GO term clusters based on the χ^2^ values for each AAR-cluster association (fig. 3*B*), which highlighted several hotspots in the heat map (white boxes). For instance, polyA, polyG, and polyA+G AARs are highly enriched in proteins involved in the development of the skeletal, nervous and endocrine systems (boxes 1–2). PolyP, polyP+R and other polyR combinations are instead particularly enriched among those regulating the development of the urinary and reproductive systems (box 3).

Similar preferential AARs enrichments were observed in protein sets controlling the development of specific parts/organs of the major anatomical system (fig. 3*C*). Thus, proteins containing polyQ repeats overall, and in certain combinations with other AARs (i.e., poly-A/G/P/S; box 1), are more associated with the forebrain, like proteins bearing polyA+polyG or polyH+polyP. Combinations of polyA/G/P repeats are instead more frequently encountered in proteins related to the hindbrain and cerebellum (box 2). Proteins involved in the development of the spinal cord and autonomic nervous system more frequently contain polyS repeats in combination with others (box 3), whereas polyH proteins are also more frequently associated with the development of the peripheral nervous system (box 4).

To obtain a finer mapping of the relative specificity and combinatorial nature of these preferential associations between AARs and anatomical (sub)systems, we generated networks in which nodes represent AARs and GO term (sub)clusters connected by edges whose thickness is proportional to the χ^2^ value of each AAR-(sub)cluster association (fig. 3*D* and *E*, supplementary fig. S4, Supplementary Material online, and supplementary table S3, Supplementary Material online).

The analysis of these data revealed four main features of the AAR functional associations.

First, protein sets regulating the development of certain systems (e.g., nervous system) are considerably more enriched with AARs and their combinations than protein sets associated with other systems (e.g., cardiovascular system).

Second, some AARs are more broadly overrepresented in protein sets related to the development of multiple anatomical (sub)systems (e.g., polyA, polyG), while others appear to be involved in more limited functional associations with specific protein sets (e.g., polyQ, polyP, polyH).

Third, many associations of AARs with specific anatomical systems are combination-dependent. Thus, combinations of one same AAR with other AARs can be either positive or negative predictors of the association with a certain anatomical system. For example, polyA or polyP repeats are much more significantly associated with the nervous system when they are combined with polyG, while the opposite is true when they are combined with polyE.

Fourth, a combination of two AARs (e.g., polyG+polyP) can be significantly overrepresented in protein sets associated with the development of a certain (sub)system (e.g., endocrine), even when the two AARs individually are not significantly overrepresented in the same proteins.

Taken together, these findings indicate that AARs in the human proteome have preferential, combinatorial associations with protein sets involved in the development of specific anatomical systems and/or their parts/organs.

### Evolutionary Dynamics of the Associations between AARs, Their Combinations, and DP Functions

These findings raised the questions whether the combinatorial associations of AARs with subsets of DPs controlling the development of specific systems/organs that we observed in the human proteome, are phylogenetically conserved, and whether they can vary quantitatively throughout phylogenesis.

**Fig. 4.**
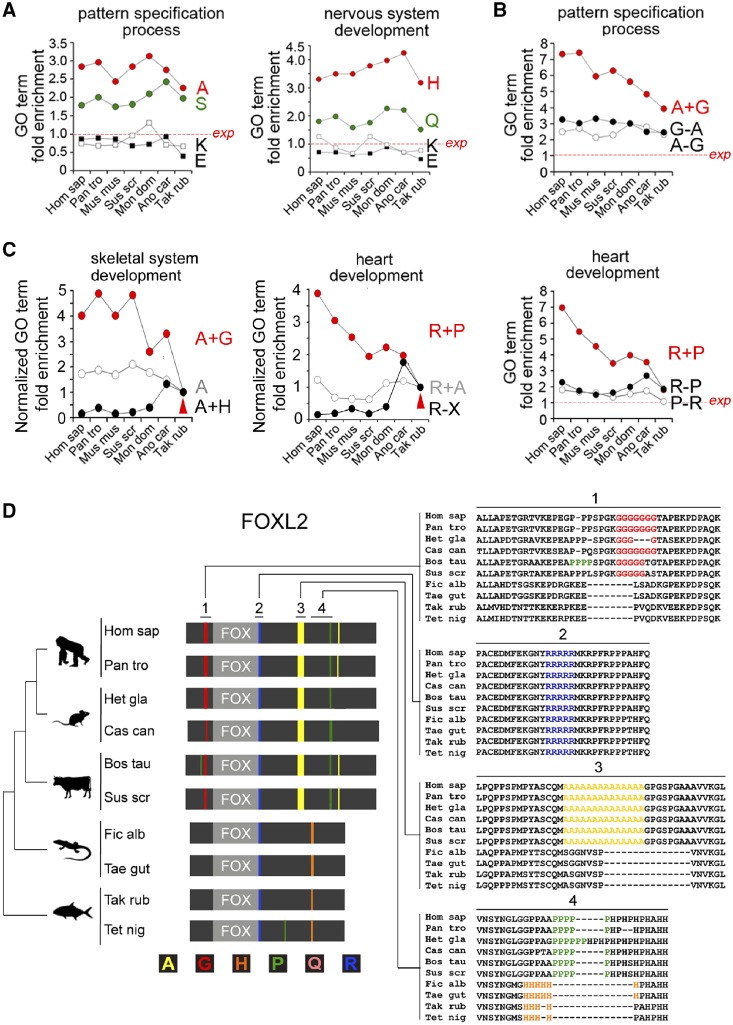
—Dynamically evolving associations of AARs with DP functions. (*A–C*) Graphs showing the relative fold enrichment throughout vertebrate phylogenesis of the indicated GO terms in the protein groups containing the indicated AARs, or their combinations, in the species on the X axis, ranging from *H. sapiens* (Hom sap) to *T. rubripes* (Tak rub). Other species name abbreviations are indicated in the Materials and Methods section. (*A*) The GO terms “pattern specification process” and “nervous system development” are stably overrepresented throughout vertebrate phylogenesis in protein groups containing certain AARs (e.g., polyA/H/Q/S) but not others (e.g., polyE/K). (*B*) Graphs showing the fold enrichment throughout vertebrate phylogenesis of the GO terms “skeletal system development” (upper) and “heart development” (lower) in proteins containing polyA±G or polyR±P, respectively. (*C*) Graphs similar to those in (*B*), showing from *Takifugu* to *Homo* the progressively increasing overrepresentation of the GO terms “skeletal system development” and “heart development” in the polyA+G and polyP+R protein groups, respectively, in comparison with other protein groups bearing the same repeats as such or in other combinations. Values are normalized to *Tak rub* (arrowhead). (*D*) Schematic representation (gray bars) of the FOXL2 protein and of some of its orthologs in species belonging to major vertebrate taxa (i.e., Primates, Rodentia, Laurasiatheria, Sauropsida, and fishes). AARs are represented by colored bars according to the color coding below the bars. The FOX domains are represented in light gray. Regions of interest (marked as 1, 2, 3, 4) of the amino acid sequence alignment of the orthologs are shown on the right.

To address these questions, we first calculated the relative AAR enrichment in protein sets associated with five major developmental GO terms (“embryo development,” “pattern specification process,” “skeletal system development,” “nervous system development,” “heart development”) and of two nondevelopmental GO terms (“carbohydrate metabolic process,” “lipid metabolic process”) in *Homo sapiens* and in seven other species representative of major vertebrate taxa that diverged from Primates at progressively more distant times (from rodents to fishes; fig. 4, supplementary fig. S4, Supplementary Material online, and supplementary table S4, Supplementary Material online).

This analysis showed that some associations between AARs and DP functions are very ancient in the vertebrate lineage. In fact, in many instances the overrepresentation of a given GO term in association with proteins containing a certain AAR can be observed from fishes to humans. Thus, for instance, polyA and polyS AARs are stably overrepresented (two to three times more than expected, *P* < 0.001 in all instances, χ^2^ test) in protein sets related to “pattern specification process” (fig. 4*A*, left panel), and polyQ/polyH AARs are overrepresented (approximately two to four times) in proteins related to “nervous system development” (fig. 4*A*, right panel; *P* < 0.01 in all instances). These enrichments are both AAR- and GO term-specific. Thus, polyE and polyK proteins are underrepresented in proteins sets associated with the same two GO terms (fig. 4*A*), and, for instance, polyA repeats are overrepresented in proteins related to “pattern specification process” and “embryo development” but not in protein sets related to “carbohydrate metabolism” and “lipid metabolism” (supplementary fig. S5*A*, left panel, Supplementary Material online).

However, in other cases (fig. S5*A*, right panel, Supplementary Material online and supplementary table S4, Supplementary Material online), the strength of several AAR/GO term associations varied progressively with evolutionary distances in vertebrate phylogenesis, as for the overrepresentation of polyG repeats in proteins related to “pattern specification process” and “embryo development.” Again, these evolutionary dynamics are GO term-specific, as they are not observed for either “carbohydrate metabolic process” or “lipid metabolic process.”

Strikingly, in many cases, changes in the strength of the AAR/GO term associations are specific to AAR combinations rather than individual AARs. Thus, the combination of polyA+polyG repeats (A + G) is increasingly more associated to “pattern specification process” from *Takifugu rubripes* to *H.sapiens*, and this increase far exceeds what found for proteins bearing polyA but not polyG (A-G), or vice versa (G-A) (fig. 4*B*). Similar dynamics are detectable for the polyR+polyP combination and “heart development” (fig. 4*C*, right panel) and the polyA+polyG combination and “nervous system development” (supplementary fig. S5*B*, left panel, Supplementary Material online).

Also in nonhuman proteomes, some AAR combinations are strong positive or negative predictors of functional associations of proteins and of their evolutionary dynamics. For example, polyA proteins are much more likely to be associated with “pattern specification process” and “nervous system development” when they do not contain also polyE repeats (A-E groups) than when they do (A + E groups) (supplementary fig. S5*B* and *C*, right panels, Supplementary Material online). The combination of polyA with polyG is progressively more associated with “skeletal system development” from *Takifugu* to *Homo*, while the opposite is true when polyA is combined with polyH (fig. 4*C*, left panel). Similar dynamics can be observed for other AAR combinations and their functional associations (fig. 4*C*, right panel and supplementary fig. S5*D*, *F*, and *H*, Supplementary Material online).

These proteome-wide dynamics could be tracked down to the evolutionary history of individual DPs, which revealed a complex variety of phylogenetic AAR dynamics across the orthologs of one same protein (fig. 4*D*, supplementary figs. S5*D*–*I* and S6, Supplementary Material online, and supplementary table S5, Supplementary Material online). Thus, in the orthologs of proteins such as FOXL2, HOXD9, and SOX1, multiple AARs can variably appear, disappear, or be stably maintained throughout phylogenesis with complex protein-specific patterns.

Taken together, these results indicate that many associations observed in the human proteome between AARs, or their combinations, and DP functions are phylogenetically ancient. In quantitative terms, these associations can either be relatively stable or evolve dynamically throughout phylogenesis with discernible trends. Traces of these proteome-wide dynamics are clearly visible in the evolutionary history of the AARs in the orthologs of individual DPs. Importantly, some of these trends of variation in the strength of AAR/DP function associations are specifically related to AAR combinations, rather than AARs as such.

### The Evolutionary Dynamics of AARs Are Interrelated and Carry Phylogenetic Signal

Given the dynamically evolving association between AARs and DP functions, it is conceivable that changes in AAR occurrence in DPs and their proteome-wide interactomes may have changed the organization of developmental PPI networks, possibly contributing to taxonomic divergence. Given the combinatorial nature of the association of AARs with developmental processes, such evolutionary rearrangements of AARs of different amino acids may have been interrelated, consistent with earlier findings on the evolution of polyQ and polyA repeats (Pelassa et al. 2014).

**Fig. 5.**
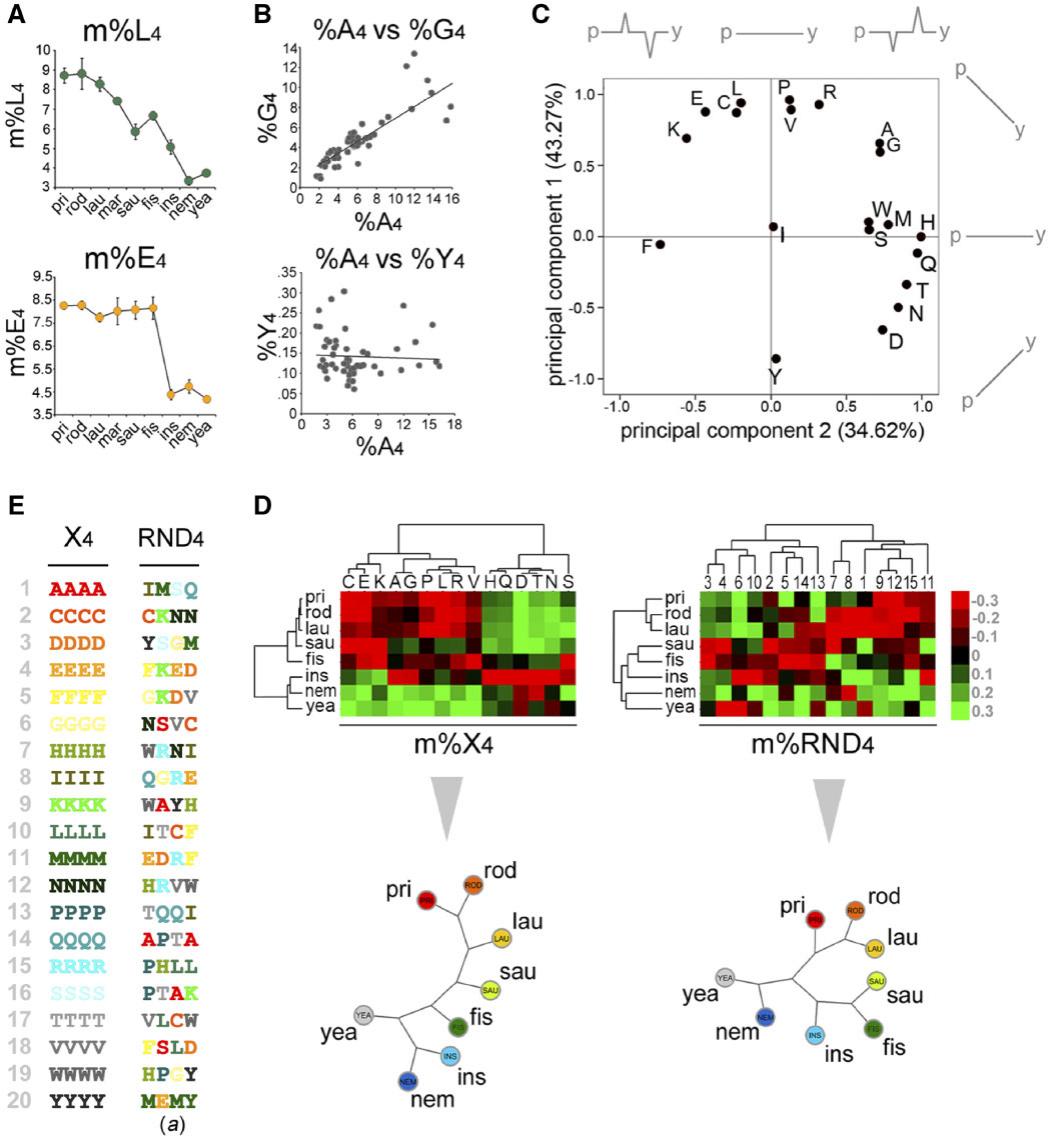
—Interrelated evolutionary dynamics of AARs carry phylogenetic signal. (*A*) Graphs showing the mean %X_4_s (i.e., m%X_4_s) in the indicated taxa for the indicated AARs. The upper and lower graphs show the phylogenetic trends of m%L_4_ and m%E_4_, respectively. (*B*) Scatterplots showing the correlation between %A_4_ and %G_4_ (upper graph)_,_ but not between %A_4_ and %Y_4_, in the 55 proteomes that were analyzed. (*C*) Graph showing the results of a PCA of the phylogenetic occurrence profiles of the 20 AARs in eukaryotic taxa. PCA identifies two major components to the variation of AAR occurrence from yeast (*y*) to humans (*h*), as schematized near two axes, each accounting for the indicated percentage of the total variance (in brackets). (*D*) Upper row. Cluster analysis of the m%X_4_ (left) and of one set of the %RND_4_ parameters (right) in the indicated taxa. Lower row. Unrooted phylogenetic trees derived from the dendrograms in the upper row. The tree derived from m%X_4_ parameters (left) reproduces standard phylogeny. This is not the case for the tree derived from m%RND_4_ parameters (right). (*E*). Primary sequence of a set of random tetrapeptides (RND_4_) obtained by reshuffling of the 20 homopolymeric peptides (X_4_) shown on the left.

To identify traces of overall, interrelated phylogenetic changes in AAR occurrence/combination that may have had evolutionary relevance, we sought to reconstruct the phylogenetic history of AARs in proteomes of major taxa, using an analytical approach that we developed for studying the evolution of polyQ and polyA repeats (Pelassa et al. 2014; fig. 5 and supplementary figs. S7 and S8, Supplementary Material online).

Thus, we analyzed the percent occurrence of proteins containing repeats of at least four units of one amino acid in reference proteomes of 55 species belonging to major eukaryotic taxa (supplementary table S1, Supplementary Material online) that is, yeast (*yea*), nematodes (*nem*), insects (*ins*), fishes (*fis*), birds and reptiles (Sauropsida, *sau*), metatherian (marsupials, *mar*) and eutherian mammals belonging to Laurasiatheria (*lau*), Rodentia (*rod*), and Primates (*pri*; individual species are listed in the Materials and Methods section). We indicate with %X_4_ each one of these percentages, where X is one of the 20 amino acids, and with m%X_4_ the mean %X_4_ in multiple species of one taxon. This analysis revealed distinctive phylogenetic trends in the occurrence of the 20 AAR types (fig. 5*A* and supplementary fig. S7*A* and *B*, Supplementary Material online). Although the occurrence of some AARs varies quite monotonically with phylogenetic distances (e.g., m%L_4_; fig. 5*A*, upper panel) from primates, others show marked increases only in specific taxa (e.g., m%H_4_), and many have intermediate features between these clock-like and taxon-specific trends (e.g., %G_4_). Notably, changes in the occurrence of certain repeats sharply mark evolutionary transitions. Thus, %E_4_ has a neat biphasic profile marking the vertebrate/invertebrate divide (fig. 5*A*, lower panel). These trends persist when normalizing the data for the amino acid usage in each proteome, showing they do not derive from changes in amino acid content (supplementary fig. S7*C*, Supplementary Material online). Strikingly, the occurrences of some repeats covary as for %A_4_ and %G_4_, or %H_4_ and %Q_4_, which have parallel phylogenetic profiles (fig. 5*B* and supplementary fig. S7*B*, Supplementary Material online), indicating that the dynamics of different AARs are interrelated throughout phylogenesis.

A PCA confirmed these results and revealed two major components to %X_4_ variation, accounting together for 77.89% of the total variance (fig. 5*C*). The first component captures the overall tendency of AAR occurrence profiles to increase or decrease with evolutionary distances from primates (*p*) to yeast (*y*), whereas the second describes the tendency of AAR trends to display taxon-specific peaks. Strikingly, the data points of the 20 AARs have a quasi-circular distribution, indicating that AAR occurrences mostly vary according to graded combinations of the two main principal components.

A cluster analysis of the m%X_4_s further confirmed these findings. This analysis revealed two major clusters of covarying %X_4_s (supplementary fig. S8*A*, Supplementary Material online) and correctly clustered the taxa in vertebrates versus invertebrates. Furthermore, when rare, less correlated repeats (average overall occurrence <0.5%, correlation <0.75) and one taxon with a limited number of analyzed species (<3, i.e., *mar*) were excluded to limit variability, the dendrogram reproduced an unrooted phylogenetic tree recapitulating the correct relationships between taxa (fig. 5*D*, left panel). These findings showed that AAR dynamics carry phylogenetic signal. This signal is considerably stronger than that carried by control sets of random, nonhomopolymeric, tetrapeptides (RND_4_; fig. 5*D* and *E*, right panels and supplementary fig. S8*B–E*, Supplementary Material online) that were generated either by reshuffling the homopolymeric X_4_ tetrapeptides (sets *a*–*e*), thus retaining the same overall amino acid composition of the homopolymeric tetrapeptides, or by adding three random amino acids after each one of the 20 amino acids (*f–j*), as a further control with a completely randomized amino acid composition.

These findings revealed that the variations in the occurrence of the 20 AARs throughout phylogenesis are highly interrelated and carry a phylogenetic signal strong enough to detect the lineage relationships between major vertebrate and invertebrate taxa.

### Phylogenetic Dynamics of AARs and Their Combinations Encode a System of Evolutionary Markers

**Fig. 6.**
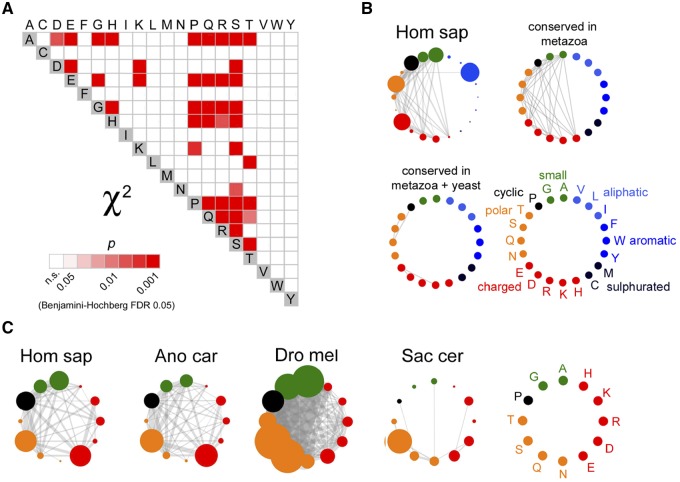
—Nonrandom pairwise combinations of AARs throughout phylogenesis. (*A*) Grid diagram showing the statistically significant pairwise AAR combinations in the human proteome that is, those combinations whose occurrence exceeds what expected by chance based on the proportions of proteins bearing the two AARs, as determined by the χ^2^ test (with Benjamini-Hochberg correction, FDR = 0.05). The significance level of each combination is highlighted in shades of red. (B) The upper left graph shows the significant pairwise combinations of AARs in the human proteome as lines connecting nodes that represent the 20 AARs, ordered based on their biochemical features as indicated in the legend (bottom right). The size of each node is proportional to the percentage of proteins containing the corresponding AAR in the proteome (%X_4_). The upper right graph shows AAR combinations that are significant also in other five metazoan species (*B. taurus*, Bos tau, *A. carolinensis*, Ano car, *T. rubripes*, Tak rub, *D. melanogaster*, Dro mel, *C. elegans*, Cae ele; top right graph). Only a few combinations are also conserved in yeast (*S. cerevisiae*, Sac cer; bottom left). (*C*) The first graph on the left, is a simplified version of the graph shown in (*B*) (Hom sap) after removing the AARs of aliphatic/aromatic/sulphurated amino acids (legend on the right). Edge tickness is proportional to the χ^2^ value for each significant AAR combination. The other graphs represent the significant AAR combinations in the indicated species.

Next, we extended the evolutionary analysis to AAR combinations. Toward this aim, we preliminarily analyzed the combinatorial landscape of AARs in species of representative eukaryotic taxa (fig. 6 and supplementary fig. S9, Supplementary Material online).

In the human proteome, the occurrence of proteins bearing certain pairwise AAR combinations (e.g., polyA+polyG) significantly exceeds what expected by chance given the number of proteins bearing either one of the two repeats in the proteome. Of the 190 possible pairwise combinations of the 20 AARs, 40 are significantly overrepresented (*P* < 0.01 in all instances, χ^2^ test, FDR 0.05; fig. 6*A*). Thirty-nine of these involve polar/charged/cyclic AARs, while only one significant association involves one hydrophobic AAR. Moreover, 27 of them are between those AARs more represented in DPs (A/E/G/H/P/Q/R/S). A large proportion of the significant combinations found in *Homo* are also significant in five metazoan species and a core set of them also in yeast (fig. 6*B*). However, the strength of these AAR associations varies dynamically throughout phylogenesis. Notably, the strength and numerosity of the combination of a given AAR with other AARs is not related to its absolute occurrence in the proteome (fig. 6C and supplementary fig. S9*A* and *B*, Supplementary Material online).

**Fig. 7.**
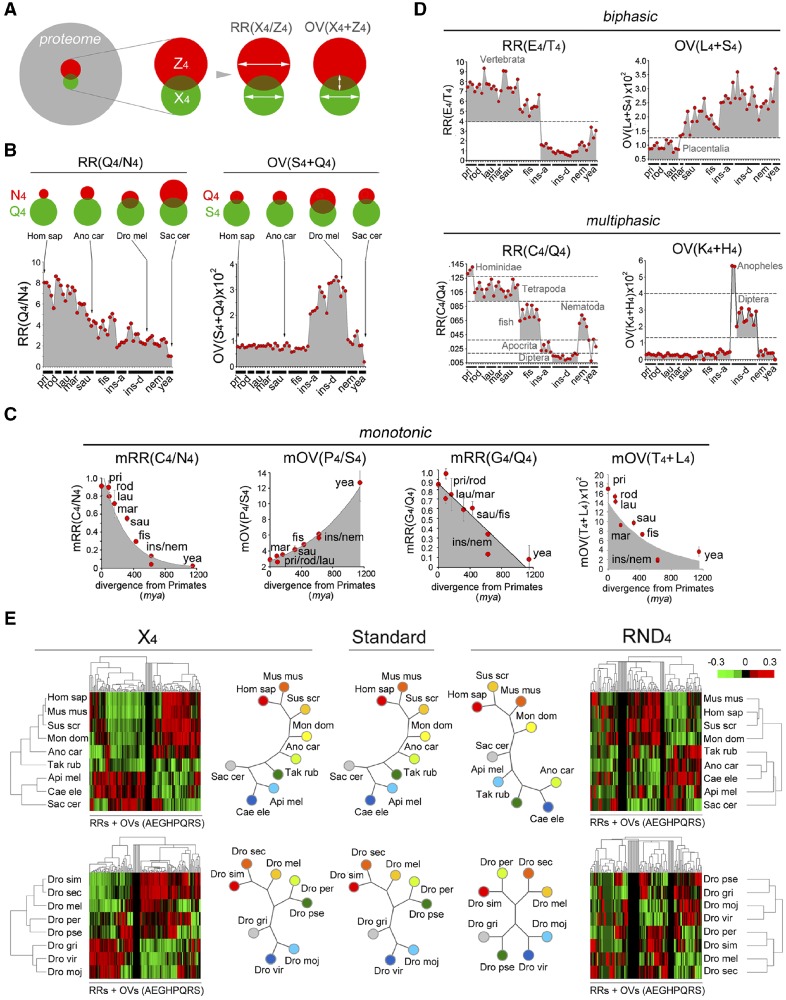
—Compound variation and combination trends of AARs encode evolutionary markers. (*A*) Venn diagrams representing how RR and OV parameters are calculated in each proteome. The large gray circle represents a given proteome and the red and green circles represent sets of proteins bearing two different AARs, X_4_ and Z_4_. The overlap area between the two circles represents proteins bearing both repeats. The RR(X_4_/Z_4_) parameter is calculated by dividing %X_4_ by %Z4. The OV(X_4_+Z_4_) parameter is calculated as the proportion of proteins containing a X_4_ repeat that also contain Z_4_ normalized to %X_4_ (Pelassa et al. 2014). (*B*) Sample plots of RR and OV parameters in individual species of the indicated taxa. Ins-a and ins-d indicates Apocrita and Diptera insects, respectively. Red dots indicate individual species from *Homo* to *Saccharomyces* (listed in the Materials and Methods section in the same order as they appear in the graphs). Light gray highlights groups of species for which the indicated parameters exceed 0. Sample Venn diagrams for selected species, graphically normalized to the size of the green circle, are shown above the graphs. (*C*) Graphs of the mean RR and OV parameters (± SEM), in the indicated taxa, that vary with monotonic trends (highlighted by trendlines and gray shading) with increasing divergence times from Primates. (*D*) Upper row. Graphs plotting RR and OV parameters with biphasic evolutionary trends that is, in which a given threshold value of the parameter identifies one or more taxa. Light gray highlights groups of species for which the indicated parameters exceed 0 or the thresholds indicated by dashed lines. Lower row. Graphs showing RR and OV parameters with multiphasic evolutionary trends. (*E*) Upper row. Cluster analyses of the RR and OV parameters derived from A/E/G/H/P/Q/R/S AARs (%X_4_) or from the corresponding control RND_4_ tetrapeptides, in the indicated species from *Saccharomyces* to *Homo*. Unrooted phylogenetic trees derived from the dendrograms of the cluster analyses are reproduced in the central diagrams, together with the unrooted tree reproducing the standard phylogeny. Note how the trees derived from X_4_ parameters reproduce the known phylogenetic relationships between the indicated species. Lower row. Same analysis as in the upper row for *Drosophila* species.

Next, we undertook a quantitative analysis of the overall phylogenetic dynamics of the pairwise AAR combinations in 55 eukaryotic species, following previously defined methodologies (Pelassa et al. 2014; fig. 7 and supplementary figs. S9 and S10, Supplementary Material online; see Materials and Methods section). Thus, in each species, for each one the 190 possible AAR combinations, we calculated two parameters. The first one, is an index of the relative occurrence of two AARs that is, the ratio between the percent occurrence of proteins bearing either one of the two AARs of that combination (e.g., %Q_4_/%A_4_) in the whole proteome that is, the “repeat ratio” (e.g., RR[Q_4_/A_4_]). The other expresses the co-occurrence of the two AARs in the same proteins (e.g., Q_4_+A_4_ proteins), as the normalized overlap between the Q_4_ and the A_4_ protein groups (e.g., OV[A_4_+Q_4_]; see Materials and Methods section and Pelassa et al. 2014; mRR and mOV indicate, respectively, the mean RR and OV values across different species in one taxon; fig. 7*A* and *B*).

We found that RR and OV parameters display a surprising variety of linear and nonlinear phylogenetic profiles (fig. 7*B*–*D* and supplementary fig. S9*C*–*E*, Supplementary Material online). Some vary monotonically, with increasing divergence times from primates, as clock-like parameters (fig. 7*C*, left panel; supplementary fig. S9*C*, Supplementary Material online). Other RRs and OVs have instead neatly biphasic profiles, and threshold values of these parameters mark transitions between taxa (fig. 7*D*, upper panel). Thus, threshold values of some RRs (e.g., RR[E_4_/T_4_] = 4) discriminate broadly vertebrates (>4) from invertebrates (<4), while thresholds of other RR/OV parameters identify more specific phylogenetic boundaries, even between closely related species (supplementary fig. S9*D–E*, Supplementary Material online**)**. Interestingly, several RRs and OVs display a multiphasic behavior, and multiple threshold values of these parameters identify multiple taxa (fig. 7*D*, lower panel and supplementary fig. S9*D*, Supplementary Material online).

Thus, RR and OV parameters overall encode a variegated system of phylogenetic markers identifying specific taxa (biphasic, multiphasic parameters) or correlating with divergence times (monotonic parameters; fig. 7*C*, left panel and supplementary fig. S9*C*, Supplementary Material online). Based on these observations, we analyzed the overall phylogenetic signal carried by the RR and OV parameters, which could be potentially stronger than that carried by the 20 %X_4_ parameters alone (fig. 7*E* and supplementary fig. S10, Supplementary Material online).

A cluster analysis of the mRRs and mOVs in higher-order taxa (*pri*, *rod*, *lau*, *mar*, *sau*, *fis*, *ins*, *nem*, and *yea*), correctly grouped them according to their actual phylogetic relationships (supplementary fig. S10*A* and *B*, Supplementary Material online), as we had found for the m%X_4_ parameters. Moreover, the same set of OV and RR parameters was sufficient to reconstruct phylogenetic relationships between individual species of these taxa (supplementary fig. S10*C*, upper row, Supplementary Material online), and even between species within lower order taxa of vertebrates (Primates; supplementary fig. S10*C*, middle row, Supplementary Material online) or invertebrates (*Drosophila*; supplementary fig. S10*C*, lower row, Supplementary Material online), except for the finer relationships between the closely related *D. grimshawi*, *D. mojavensis*, and *D. virilis*.

This phylogenetic signal further improved using a limited subset of RR and OV parameters (128 over 800) resulting from polyA/E/G/H/P/Q/R/S that is, those AARs most represented in DPs. Using this approach, the phylogenetic trees encompassing all taxa from yeast to humans, as well as the primate and Drosophila trees were all correctly solved (fig. 7*E* and supplementary fig. S10*D*, Supplementary Material online). As for the %X4 parameters, the phylogenetic signal carried by RR+OV parameters was much stronger for AARs than for control random tetrapeptides (RND_4_; 100% vs 20% correct trees, *P* < 0.01, FE test).

Together with our previous findings, these observations indicate that quantitative changes in the relative occurrence (RRs) and in the combinatorial patterns of cooccurrence (OVs) of AARs, especially of those enriched in DPs, mark taxonomic differences throughout phylogenesis even at the level of closely related species.

## Discussion

The results of our analyses indicate that AARs in DPs, DP interactomes, and proteomes display nonrandom combination patterns, functional associations, and interrelated phylogenetic dynamics. The observed combinatorial distribution and evolution of AARs in proteomes configure a system of markers of DP functions and evolutionary transitions, consistent with a generalized role of AARs as a whole system of regulatory sequences in developmental processes with evolutionary implications. These findings are novel and establish a quantitative and qualitative framework tracing the functional and evolutionary history of AARs in proteomes as a whole system of interrelated sequences, rather than sporadic functional regulators in some DPs. From this perspective, AARs appear to define a combinatorial regulatory system of specific developmental processes. Our findings also define a novel set of quantitative parameters (RR and OV) that mark evolutionary transitions.

### AARs as Combinatorial Markers of DP Functions

Our findings indicate that DPs pervasively contain AARs in variable combinations, with differential distributions across functionally distinct DP families and subfamilies, such as the anterior, central and posterior HOX genes. Also at the proteome level, we found evidence of preferential occurrences of AARs, and their combinations, in protein sets regulating specific developmental processes, ranging from embryo patterning to the morphogenesis of specific systems/organs. Overall, the differential occurrence of distinct AARs in functionally specialized DP families and subfamilies (anterior/central/posterior HOX; Gilbert and Barresi 2016) may underlie the formation of functional networks of proteins that cooperate in the development of specific systems/organs.

If previous studies observed that certain AARs have some general functional associations (Albà et al. 1999; Simon and Hancock 2009; Schaefer 2012; Radó-Trilla and Albà 2012), our analyses outline novel core elements of a combinatorial AAR-based functional code in metazoan DPs and proteomes by which specific AARs and their combinations are overrepresented in proteins directing specific aspects of development. This code may also involve still unidentified elements co-occurring with AARs, such as conventional protein/nucleic acid binding domains (Pelassa and Fiumara 2015; Erives 2017), that may confer to it even greater functional specificity.

Importantly, we also found that the association of AARs with DP functions is either relatively stable over long evolutionary periods or instead vary dynamically with detectable trends. For instance, while the polyA/“embryo patterning” association has been constantly two to three times more frequent than expected for the past ∼500 million years, the polyG/“embryo patterning” association has increased dramatically over the same time span. Strikingly, this increase can be attributed mostly to the progressive increase of the polyA+polyG combination, rather than of polyA or polyG as such, in DPs controlling embryo patterning. This is not an exception, and similar dynamics were observed for other AAR combinations. These findings are consistent with the evidence of structural and functional interactions of different AARs in one same protein, as observed for RUNX2 (polyQ/polyA) and the androgen receptor (polyQ/polyG; e.g., Fondon and Garner 2004; Bhattacharyya et al. 2006; Pelassa et al. 2014; Grigorova et al. 2017).

These functional interactions may favor the evolutionary emergence of AAR combination patterns in proteomes. Epistatic interactions between AAR-encoding mutations, or between these and single nucleotide polymorphisms (Press and Queitsch 2017), may thus be important in determining the phenotypic effects of AARs, as also suggested by disease-related observations (Gispert et al. 2012).

Different types of AARs, such as polyQ, polyA, and polyP, are increasingly recognized as structured sequences with important regulatory roles of protein interactions and function, rather than disordered, misfolding-prone spacers—as often assumed (e.g., Gemayel et al. 2010, 2015; Fiumara et al. 2010; Schaefer et al. 2012; Pelassa et al. 2014; Pelassa and Fiumara 2015; Mier et al. 2017; Chavali et al. 2017). Importantly, these AARs have been identified early on as transcriptional regulators (e.g., Gerber et al. 1994), and evidence exists that they favor the functional diversification of duplicated TFs (Radó-Trilla et al. 2015). In this respect, our findings strongly suggest that the coordinated appearance of one same AAR in functionally related DPs may have driven the emergence of novel DP interactomes and/or varied existing ones (Hancock and Simon, 2005; Pelassa and Fiumara 2015).

It is also interesting to note that the results of our functional analyses of polyA and polyQ proteins indicate that they are involved in the development of anatomical systems that correspond to those involved in the polyA and polyQ expansion diseases. In fact, polyA diseases are mostly (eight out of nine) skeletal and/or neurodevelopmental syndromes, with endocrine dysfunction in some cases (Albrecht et al. 2004), and our results show that polyA proteins are significantly associated with skeletal, nervous and endocrine development. In a similar manner, polyQ expansion diseases are neurological disorders with some muscular involvement (Zielonka et al. 2014; Lieberman 2018), and we find that polyQ proteins are especially associated with neural and muscular development.

### Compound Dynamics of AARs as Evolutionary Markers

We found extensive evidence of taxon- and species-specific changes in the relative occurrence (RR parameters) and combination (OV parameters) of AARs, which may signal overall rearrangements of DP interaction networks that contributed to the divergence of species/taxa. In fact, our analyses indicate that RR and OV parameters vary throughout phylogenesis marking a variety of taxonomic boundaries. Although some vary with monotonic trends, as clock-like parameters, many of them show distinctive taxonomic fluctuations marking specific taxa or major taxonomic divides. These compound AAR dynamics carry overall a considerable phylogenetic signal which is significantly stronger than that carried by random tetrapeptides. Notably, this signal is even stronger when considering only a subset of RR/OV parameters derived from those AARs enriched in DPs which may have a more direct influence on development.

In principle, the observed phylogenetic dynamics of AARs may be attributed purely to the occurrence of neutral AAR-encoding indel mutations and genetic drift. While these processes will certainly have contributed to at least some of the observed patterns, existing evidence indicates that the origin of AAR evolutionary dynamics conceivably lies in a complex interplay of mutational and selective forces. AARs originate and expand/contract as a result of replication slippage or unequal crossing-over (Albà et al. 1999; Sainudiin et al. 2004; Owens et al. 2013; Warren 1997), leading to the expansion/contraction of DNA triplet repeats, some of which are more slippage-prone (Kruglyak et al. 2000). While taxon-specific differences slippage rates (Canceill et al. 1999; Flores and Engels 1999; Ross et al. 2003; Laidlaw et al. 2007; Castillo-Lizardo et al. 2014), codon usage (Albà et al. 1999), unequal crossing-over (Hoffmann et al. 2008), and repair mechanisms (Sia et al. 2001) may contribute to the evolution of AARs, analyses of mutation rates and codon usage show that selective mechanisms also play significant roles in shaping their evolutionary dynamics (e.g. Hancock et al. 2001; Mularoni et al. 2010; [Bibr evz216-B52][Bibr evz216-B44]; Li et al. 2012), including convergent evolution (Lavoie et al. 2003), after they are produced by mutational processes intrinsic to genome replication (Dover 1989, 2000).

Besides their contribution to the understanding of AAR functions, our findings may potentially be useful also in phylogenetic studies by providing sets of novel, alignment-free quantitative parameters that may help to resolve ambiguities in conventional phylogenetic analyses based on sequence alignments (e.g. Haubold 2014).

### Biological Significance of the Combinatorial Functional Distribution and Interrelated Evolutionary History of AARs

The interrelated changes in the occurrence and combination of AARs that we observed throughout phylogenesis may represent evolutionary traces of AAR-mediated regulatory changes in developmental processes contributing to morphological and behavioral evolution.

In yeast, recent evidence indicates that AAR variability increases evolvability also through the rewiring of protein interactomes (Gemayel et al. 2015; Chavali et al. 2017). Our findings strongly suggest that this may also be the case for Metazoa. Indeed, we found quantitative evidence supporting this hypothesis by showing that DPs containing polyA/G/P/Q/S repeats have interactors in which the same AARs are overrepresented. These findings are consistent with the emerging roles of poly-A/-Q/-P in mediating PPIs by forming coiled coils (polyQ, polyA; Fiumara et al. 2010, 2015; Schaefer et al. 2012; Pelassa et al. 2014; Gemayel et al. 2015) and PP-II structures (polyP; Adzhubei et al. 2013). Moreover, also polyG and polyS repeats can function as protein localization signals (Wolf et al. 2013) and polyglycylation is even used as a PPI-modulating posttranslational modification (Redeker et al. 1994), consistent with a role in PPIs also for these AARs (Pelassa and Fiumara 2015; Lilliu et al. 2018). In contrast, the interactomes of DPs bearing charged repeats, which would be repulsive in homotypic interactions, did not show a similar enrichment of the same AARs, consistent with the view that these AARs mediate interactions with partners bearing an opposite charge. These may include other charged AARs, DNA, or histones (Dean 1983; Nam et al. 2001; DeRouchey et al. 2013). These electrostatic interactions may, for example, regulate the activity of TFs bearing charged AARs by modulating their binding affinity for DNA and chromatin.

In metazoa, the occurrence of specific AARs in certain proteins, like RUNX2 and POU3F2 in vertebrates and *hunchback* in *Drosophila*, have been directly related to morphological and behavioral evolution (Treier et al. 1989; Fondon and Garner 1994; Nasu et al. 2014; Hashizume et al. 2018). These and similar observations (Galant and Carroll 2002; Anan et al. 2007; O’Malley and Banks 2008; Chew et al. 2012) suggested the possibility that AARs may have important evolutionary roles by increasing phenotypic variability (Dover 1989; Haerty and Golding 2010b) as regulatory “tuning knobs” (King et al. 1997).

If AARs are tuning knobs modulating the activity of DPs, our findings indicate that they are not evolving sparsely and independently in a few regulatory proteins but in a more interrelated, combinatorial manner as on a “control panel” of knobs in functionally related DPs. Along the same metaphor, our analyses indicate that the number, interconnection, and the controlled functions of the regulatory knobs on this panel have changed throughout phylogenesis with discernible, previously unrecognized, trends. In this respect, we purposely focused on analyzing the presence/absence/combination of AARs across species (i.e., the presence/interconnection of the knobs), rather than their length variation (i.e., the degree of turning of the knobs) which allows additional levels of regulation (Gerber et al. 1994; Pelassa et al. 2014; Gemayel et al. 2015).

Thus, the structural/functional properties of AARs may ultimately underlie the modulation of the activity/interactions of DPs, consistent with an evolutionary paradigm that views mutations in the coding part of genes as important players in evolutionary processes (Hoekstra and Coyne 2007; Lynch and Wagner 2008) together with those in noncoding, *cis*-regulatory gene regions (e.g., Carroll 2008; Vinces et al. 2009). In this context, AARs are interesting because they essentially introduce regulatory modules of function in the coding part of DPs outside their DNA recognition domains, thus not altering their binding specificity.

The mutation rate of AAR-encoding repeats is considerably greater than for point mutations (Ellegren 2000), and thus the sudden elongation, contraction, or deletion of AARs in key DPs could contribute to driving relatively rapid evolutionary processes (Dover 1982; Gould 2002), as dramatically exemplified by polyA-expansion developmental diseases, in which modest AAR expansions induce macroscopic skeletal changes (Albrecht et al. 2004; Messaed and Rouleau 2009). In this view, the pathological consequences of AAR expansion may be seen as the exaggeration, or dysregulation, of the physiological structural and functional roles of these repeats (Fiumara et al. 2010; Orr 2012; Blum et al. 2013; Pelassa et al. 2014).

In conclusion, our analyses define novel quantitative evidence and a proteome-wide interpretive framework supporting the notion of a combinatorial role of AARs as a system of regulatory sequences that mark functionally related DPs, and whose interrelated evolutionary dynamics signal evolutionary distances and transitions. These findings may provide critical guidance for the informed experimental dissection of the functional roles of specific AARs, and their combinations, in evolution and development.

## Supplementary Material

Supplementary data are available at *Genome Biology and Evolution* online.

## Supplementary Material

evz216_Supplementary_Data
