# Supplementary material for: Compound Dynamics and Combinatorial Patterns of Amino Acid Repeats Encode a System of Evolutionary and Developmental Markers
Source: Genome Biol Evol. 2019 Oct 7;11(11):3159–78. doi: 10.1093/gbe/evz216 (PMC6839033; doi:10.1093/gbe/evz216)
Supplement: evz216_Supplementary_Data [file evz216_supplementary_data.pdf]

Supplemental materials

Compound dynamics and combinatorial patterns of amino acid repeats encode a system of evolutionary and developmental markers

Ilaria Pelassa[1], Marica Cibelli[1], Veronica Villeri[1], Elena Lilliu[1], Serena Vaglietti[1], Federica Olocco[1], Mirella Ghirardi[1,2], Pier Giorgio Montarolo[1,2], Davide Corà[3,4], and Ferdinando Fiumara[1,2*]

[1]Department of Neuroscience *Rita Levi Montalcini*, University of Torino, 10125 Torino, Italy

[2]National Institute of Neuroscience (INN), 10125 Torino, Italy

[3]Department of Translational Medicine, Piemonte Orientale University, Novara, Italy

[4]Center for Translational Research on Autoimmune and Allergic Disease (CAAD), Novara, Italy

**Correspondence*: ferdinando.fiumara@unito.it

SUPPLEMENTAL FIGURE LEGENDS

Figure S1 – Differential distribution of AARs and their combinations in human DPs

A. Schematic representation of selected human DPs and their AARs as in *Fig. 1A* **B.** Pie charts summarizing AAR occurrence in DPs as in Fig. 1B. **C.** Histogram showing the percentage of proteins containing the indicated AARs in 9 human DP families. **D.** Histogram showing the differential occurrence of charged AARs and non-charged (*other*) AARs of a single type (1) or of multiple types (>1), in antero-posterior *vs* central HOX proteins.

-

Figure S2 – Differential occurrence and co-occurrence of AARs in human HOX proteins

Schematic representation, as in *Fig. 1A,* of the AAR distribution in the HOX protein family. The Homeobox domain (HOX) is in *light grey*. Bar lengths are proportional to actual protein lengths.

Figure S3 – Overrepresentation of PPI-related AARs in the interactome of DPs

A. Histogram representing the overrepresentation of proteins contining a given AAR among the interactors of DPs containing the same AAR. Values are normalized to the AAR occurrence in the whole proteome. **B.** Graph of the interactome formed by polyA DPs and their polyA interactors. **C.** Graphs as in *B*, for polyG, polyP and polyS DPs.

Figure S4 – Preferential occurrence of different AARs in DPs with specific functions

A-B. Graphical representation of the strength of the statistical association between developmental GO term clusters (A, *upper rows)* and subclusters (B, *upper rows*) and AAR combinations (A-B, *lower rows*), as in *Fig. 3D,E*. Line thickness in proportional to the χ^2 value of each association. Abbreviations as in *Fig. 3D,E*. Other abbreviations: *adr*, adrenal glands; *atr*, cardiac atria; *con*, conduction system; *hea*, heart; *hhs*, hypothalamic-hypophyseal system; *mus*, cardiac

muscle; *pan*, endocrine pancreas; *pat*, parathyroid glands; *sep*, interatrial or interventricular septum; *thy*, thyroid; *val*, cardiac valves; *vas*, blood vessels; *ven*, cardiac ventricles.

Figure S5 – Dynamic, combinatorial association of AARs with DP functions

A-C. Graphs showing the relative fold enrichment throughout vertebrate phylogenesis of the indicated GO terms in the protein groups containing the indicated AARs, or their combinations, in the indicated species, as in *Fig. 4A-C*. Species name abbreviations are listed in the *Methods*. **D-I. D,F,H.** Evolutionary dynamics of the relation between the indicated AAR combinations and GO terms. These proteome-wide trends parallel what observed in the evolutionary history of individual proteins and shown in panels E,G,I. **E,G,I.** Schematic representation, as in *Fig. 4D*, of the human protein HOXD9 (*E)*, EVX2 (*G*), and SOX1 (*I*) and some of their non-human orthologs in species belonging to major taxa for which the complete sequence is available. The *high mobility group* (HMG) and *homeobox* (HOX/HX) domains are represented in *light grey*.

Figure S6 – Phylogenetic history of AARs in individual DPs

A. Graphs showing the percent proportion of the orthologs of the indicated DPs containing the indicated AARs (A/E/G/H/P/Q/R/S), among those that are available in the *pri*, *rod*, *lau*, *sau*, *fis* vertebrate taxa. Note that all the available ortholog sequences for a given protein were analyzed, including incomplete sequences reported in Ensembl. This inclusive approach may cause some of the irregularities in the occurrence trends of certain AARs across taxa owing to the apparent lack of AARs in the orthologs of certain taxa for which only incomplete sequences are available.

Figure S7 – Interrelated evolutionary dynamics of AARs carry phylogenetic signal

A. Graphs showing the percentage of proteins containing each one of the 20 AARs (i.e. %X_4s) in the indicated species. **B.** Graphs displaying the m%X_4s values (± SEM) in the different taxa. **C.** Graphs displaying m%X_4s after normalization to amino acid usage in each taxa. Data were further normalized to the average value of each parameter in the taxon *yea*.

Figure S8 – Analysis of phylogenetic signal derived from the occurrence of random tetrapeptides

A. Cluster analysis of the mX_4 parameters in the indicated taxa. **B.** Primary sequence of ten random sets of tetrapeptides (RND_4) obtained either by reshuffling (sets RND_4-*a-e*) of the 20 homopolymeric peptides (X_4) shown in *Fig. 5E* (*left* column)**,** or by selecting three random amino acids after the first amino acid of the X_4 peptides (sets RND_4-*f-j*). **C.** Graph displaying the %X_4s for the 55 species set (*upper left* graph) and the m%X_4s for the indicated taxa (*lower left* graph). The corresponding graphs for one of the sets of random tetrapeptides are on the *right*. Values in all graphs are normalized to the m%X_4s in the taxon *yea*. **D.** Graph displaying average standard deviation σ (± SEM) of the variation profiles of the 20 %X_4s across the 55 species versus the average σ of the variation profiles of the 20 %RND_4s (for each one of the ten sets *a-g*) in the same species. The average σ is significantly higher in the X_4 than in any one of the ten RND_4 groups. **E.** Cluster analyses of the *b-j* sets of %RND_4 parameters in the indicated taxa.

Figure S9 – AAR occurrence and co-occurrence profiles carry phylogenetic signal

A. Graphs of the statistically significant pairwise combinations of AARs in the human proteome (*top left*) and other 6 proteomes (species abbreviatons in the Methods section) **B**. Simplified version of the graphs shown in *C*, in which only the significant pairwise combinations of polyA repeats with other AARs are represented, as indicated in the legend (*bottom right*). **C.** Graphs of RR and OV parameters varying among the indicated species with monotonic trends (highlighted

by trendlines and gray shading) with increasing divergence times from *Homo sapiens* (*Homo*, *two left graphs*) and from *Drosophila sechellia* (*Dro sec*, *two right graphs*). **D-E.** Graphs plotting the indicated RR and OV parameters, varying with either biphasic or multiphasic profiles, as in *Fig. 7D.*

Figure S10 – Phylogenetic signal carried by RR and OV parameters

A. Heat map of a hierarchical cluster analysis of all mRR and mOV parameters *versus* major eukaryotic taxa. **B.** An unrooted phylogenetic tree (*left*) derived from the cluster analysis dendrogram in panel *A* reproduces the known phylogenetic relationships between the indicated taxa (*right*; Hedges et al., 2006). **C.** *Upper row*. The *first* and *last panels* from the left show cluster analyses of the RR and OV parameters derived from all the 20 X_4s or from the 8 X_4 subset (A/E/G/H/P/Q/R/S), respectively, in the indicated species from *Saccharomyces* to *Homo*. Unrooted phylogenetic trees derived from the two cluster analyses are reproduced in the central diagrams, together with a tree reproducing the standard phylogeny (*middle*). The *middle* and *lower rows* show the same analysis as in the *upper row* for primates and *Drosophila* species, respectively. **D.** As in panel *C*, heat maps of a hierarchical cluster analysis of mRR and mOV parameters derived from the A/E/G/H/P/Q/R/S X_4 subset, or from RND_4 subset, and the resulting unrooted phylogenetic trees of 5 primates in comparison with the standard phylogeny (*middle*).

Table S1 – *Occurrence of AARs and their pairwise combinations in 55 eukaryotic species*

Table S2 – *Manual semantic clustering of analyzed GO terms containing the word "development" associated with human proteins*

Table S3 – *χ^2 values and statistical significance after χ^2 test and Beniamini-Hochberg correction (FDR = 0.05) for each association between AARs and GO term (sub)clusters.*

Table S4 – *Evolutionary analysis of the associations between GO terms and AAR proteins*

Table S5 – *Proportion of orthologs of the 167 analyzed DPs that contain AARs in each taxon*

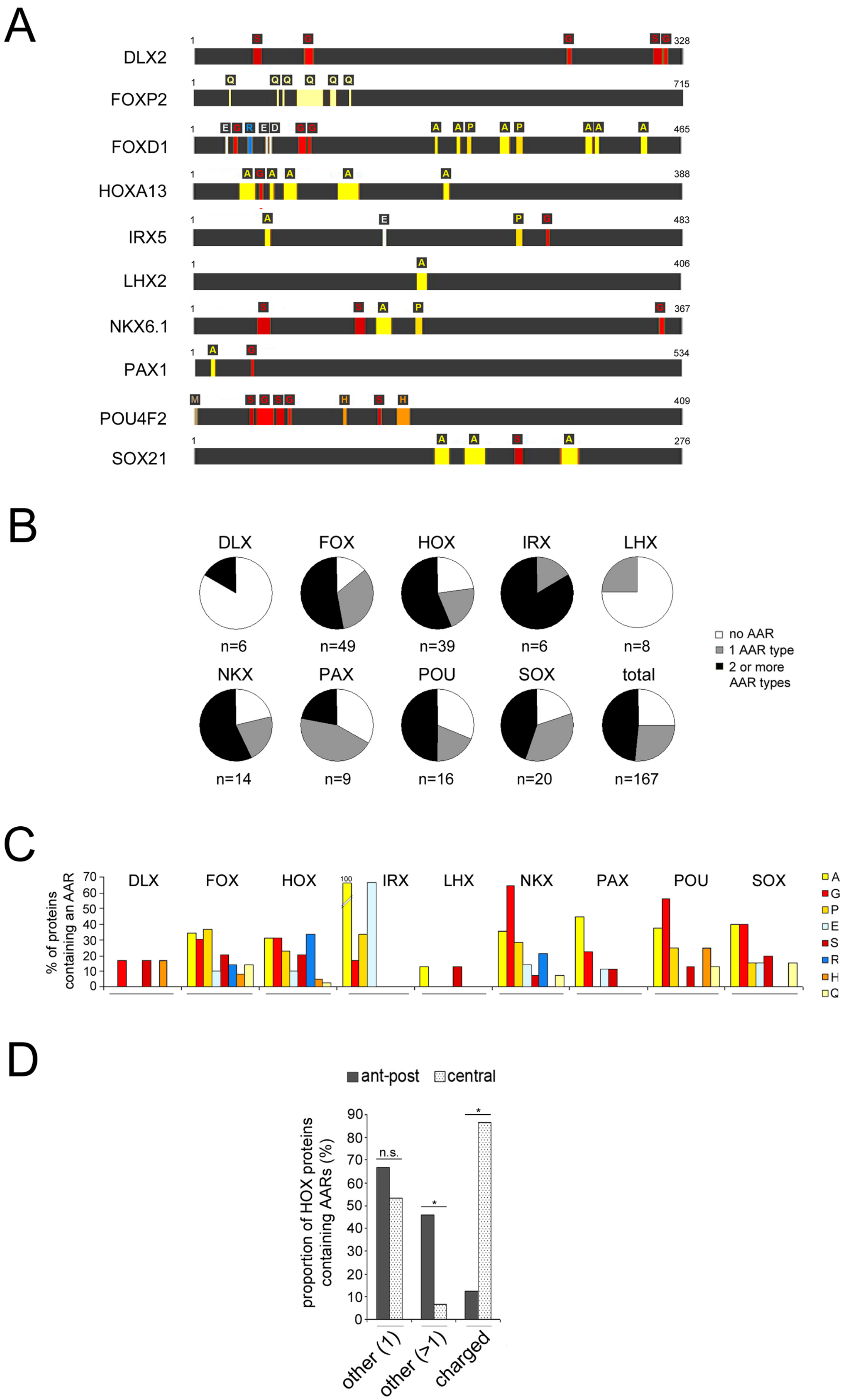

Supplemental Figure 1

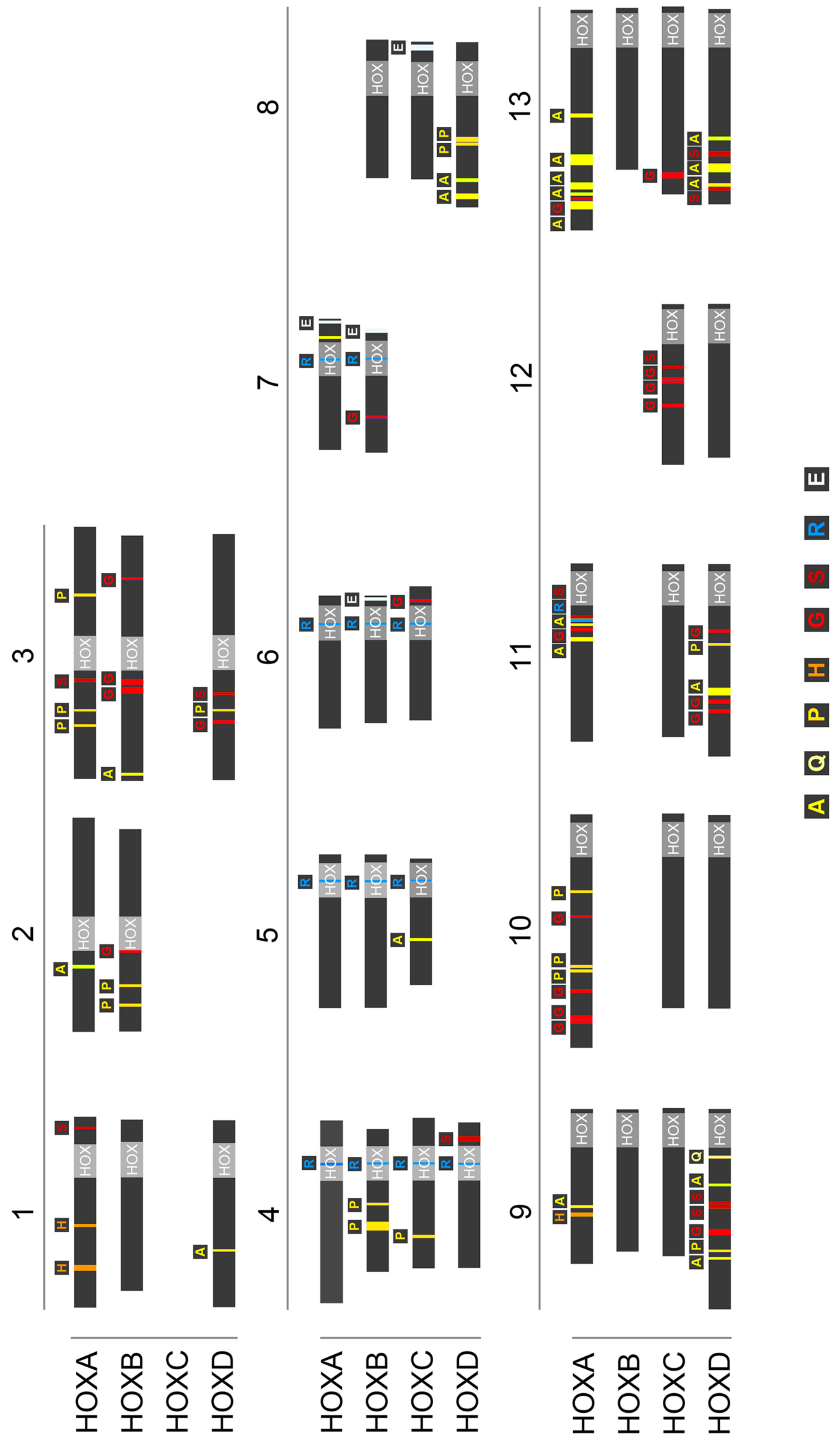

Supplemental figure 2

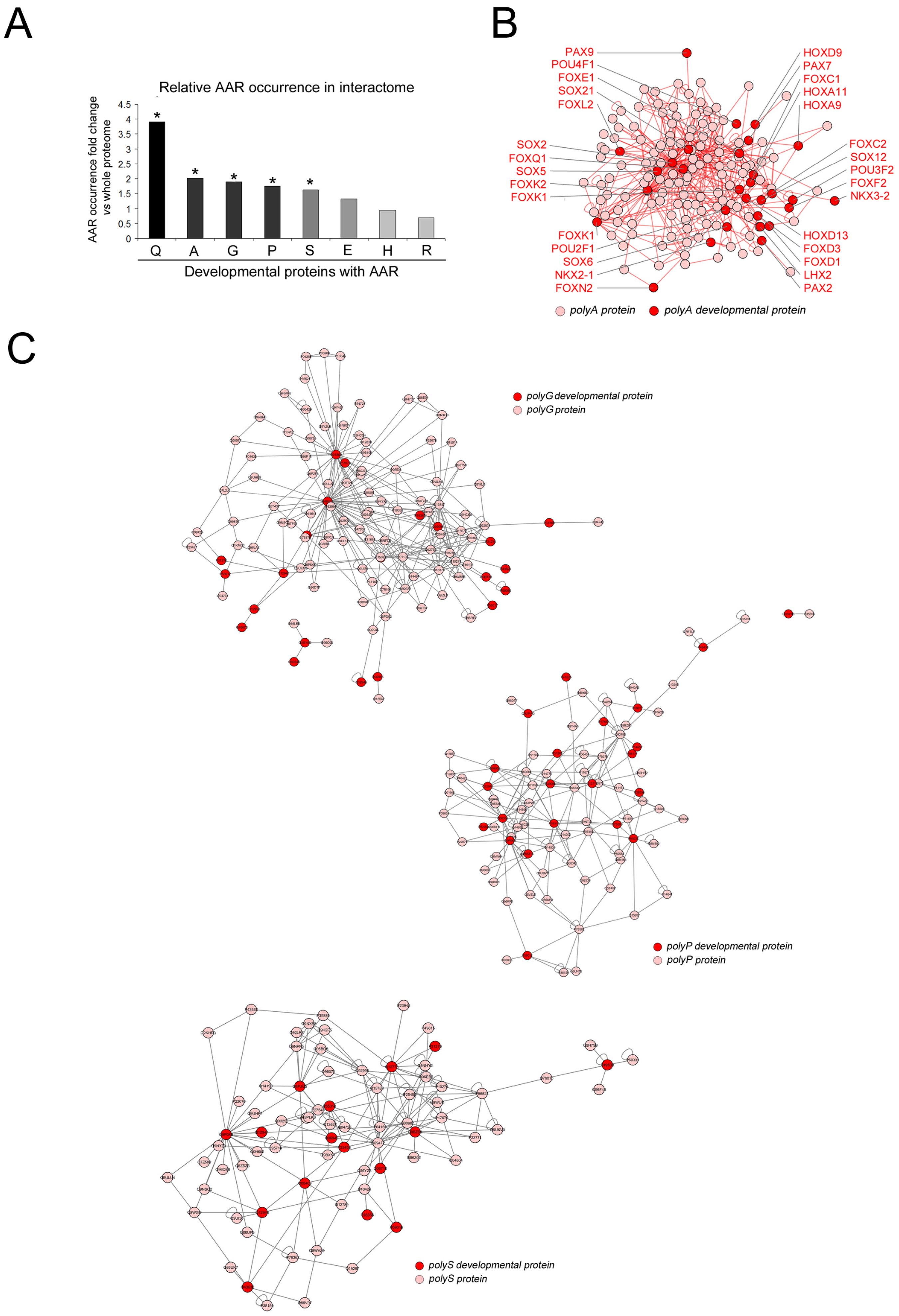

Supplemental figure 3

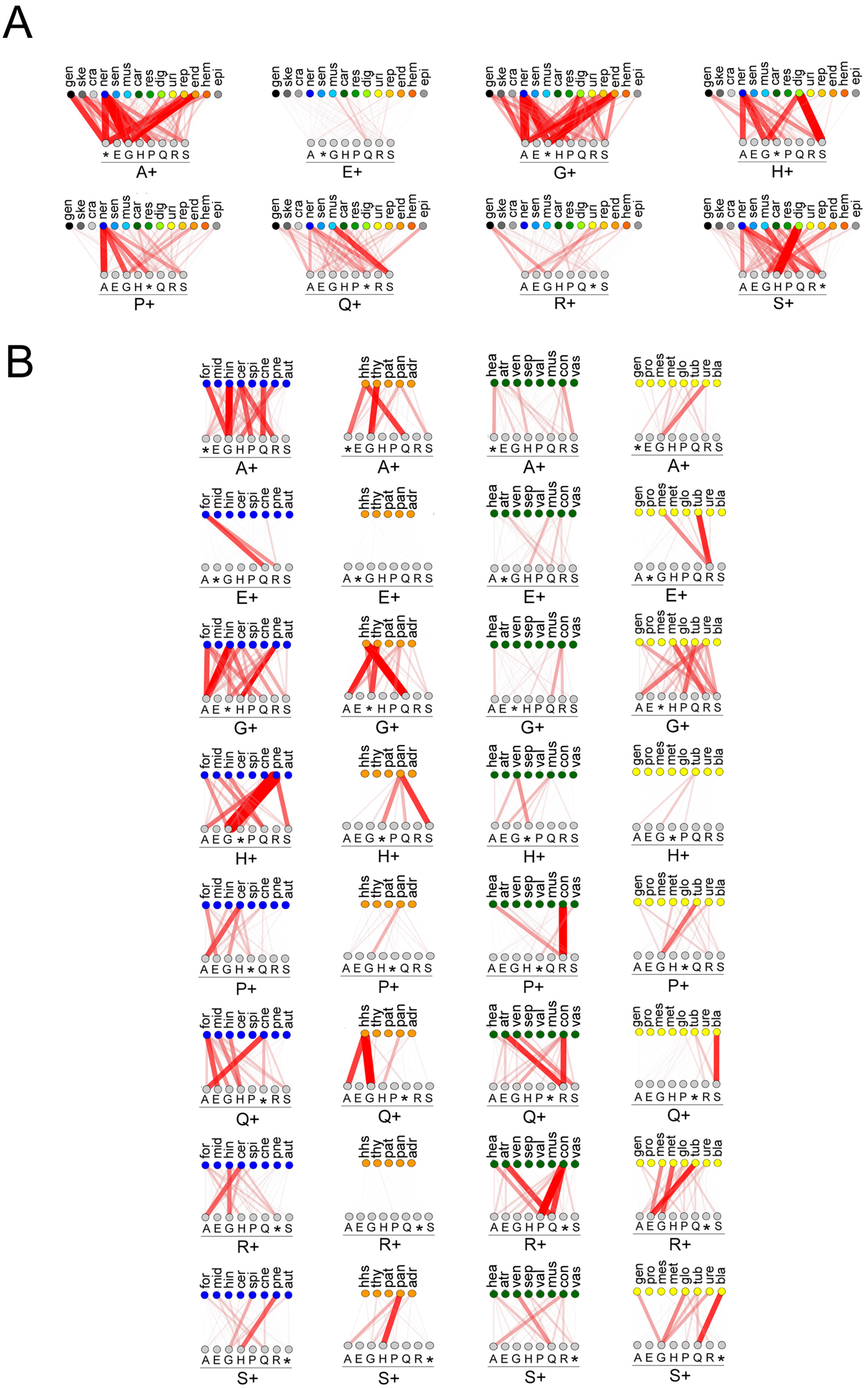

Supplemental figure 4

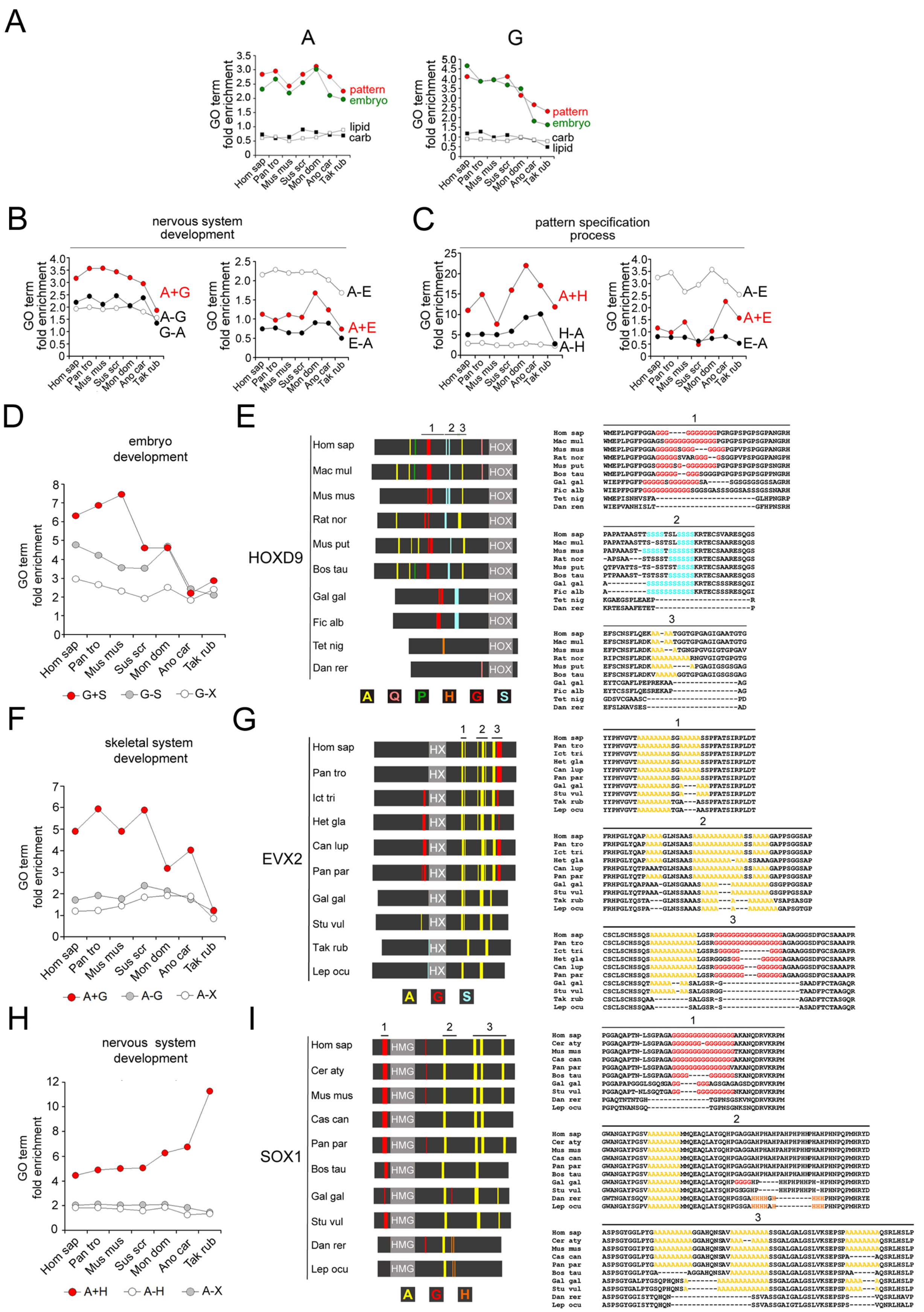

Supplemental figure 5

A

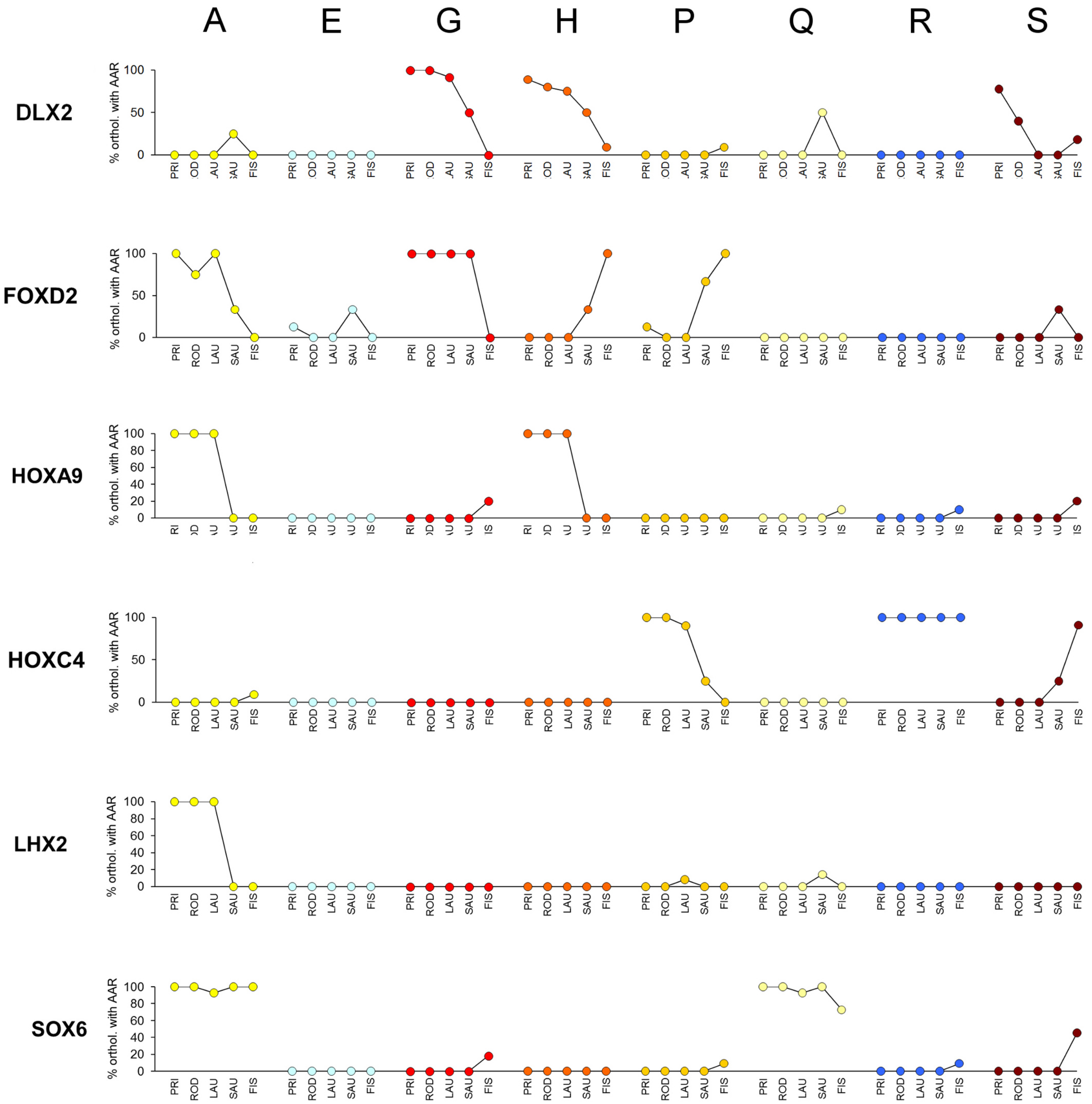

Supplemental figure 6

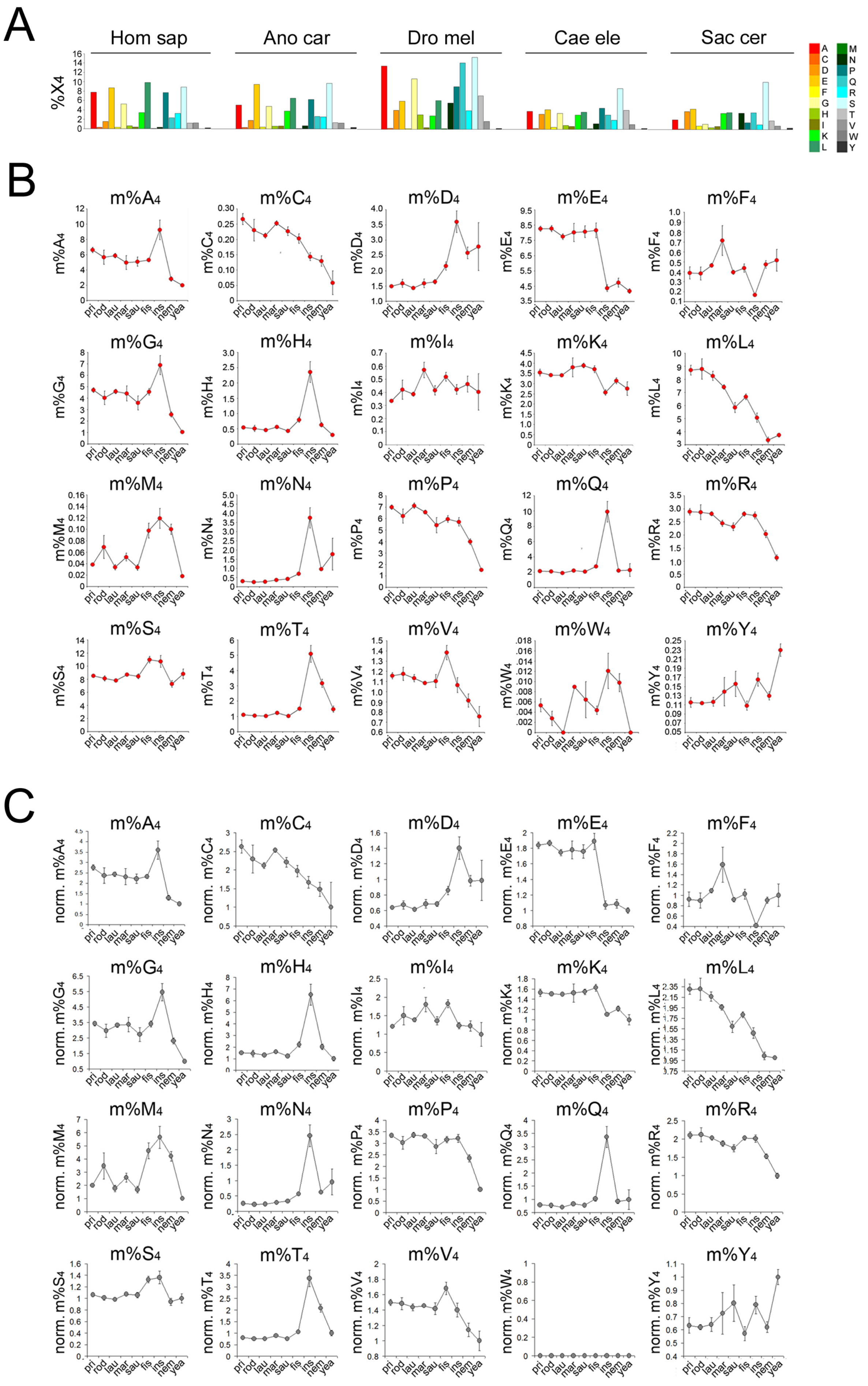

Supplemental figure 7

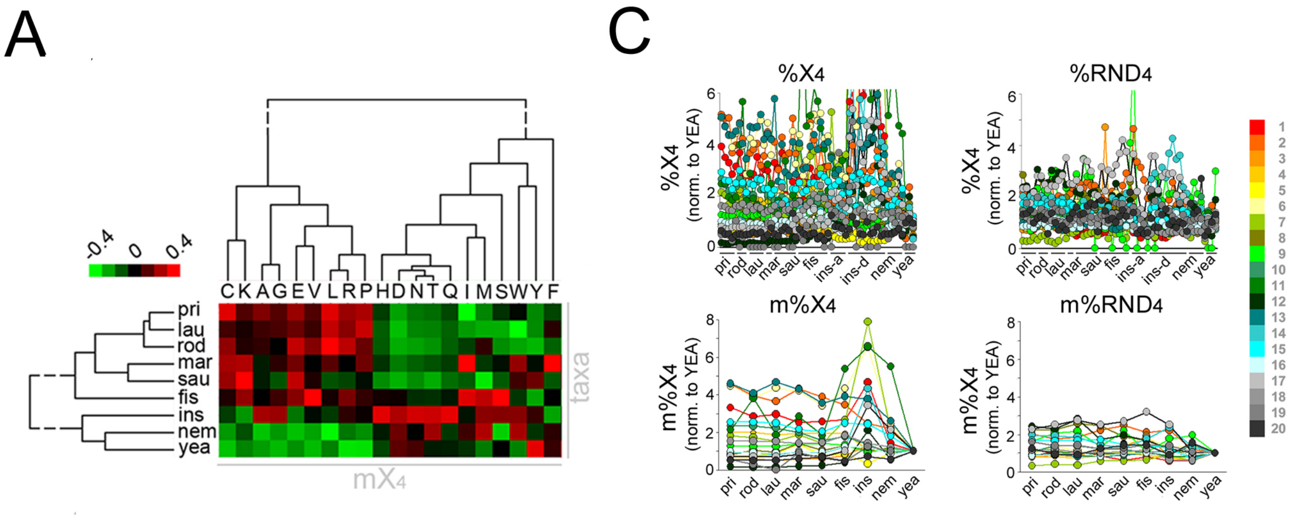

B

	RND4	RND4	RND4	RND4	RND4	RND4	RND4	RND4	RND4	RND4
1	IMSQ	IDAE	DRGG	KKGR	REMI	AYWT	AYGV	AVKD	AGFK	ATPG
2	CKNN	MHTW	VEAL	VWDA	WLKG	CLRY	CFEY	CIPS	CMIE	CHQD
3	YSGM	WFCY	ECQP	ENCL	TTFL	DPML	DEYP	DTWM	DMWG	DWPR
4	FKED	DLVM	FSNA	QWLN	CQIH	EAWH	EKYC	ENIL	EDRT	EGNL
5	GKDV	RNGS	FWYW	IFFI	MGMA	FEPV	FDKL	FRVN	FDVQ	FSPV
6	NSVC	ADCK	KDRH	RTKT	CPCS	GCDP	GSDH	GLMC	GFQN	GHNE
7	WRNI	WITK	AMPH	FCPM	DNGE	HPDN	HYFT	HSFD	HCPR	HYVL
8	QGRE	TNRF	SEQY	PVNC	GAQW	IDEA	IVWC	IEHD	IHWQ	IDKS
9	WAYH	MIWR	MFIQ	ARLS	KRHQ	KGLP	KLQH	KAYF	KATE	KFWC
0	ITCF	NPFY	PNPY	EDDN	DHRQ	LEWY	LTPD	LYNK	LMIW	LKTM
1	EDRF	YGHG	NMCG	FYMP	FVDK	MVTA	MNFA	MHVK	MKSN	MQNH
2	HRVW	GCAL	MLYW	QPTH	MNHF	NAWP	NQMK	NMAT	NRAF	NCWM
3	TQQI	MCEP	VLRD	EMGA	VLVD	PTAL	PSYN	PLHC	PAVS	PKCM
4	APTA	ATID	GKSQ	MEDV	AIPN	QPHC	QFNP	QETI	QDSE	QEYP
5	PHLL	SNPF	HTNI	YHIL	YCAP	RQSK	RWHF	RKAV	RLGA	RWKL
6	PTAK	PVSE	DKIA	GIHC	WSTP	SFVE	STKN	SRCM	SPLQ	SLAN
7	VLCW	QHYK	WITH	SYQW	ELRY	TGPE	TIAY	TIFC	TEGN	TRHP
8	FSLD	QQVR	FCVS	WASV	NKTY	VKRN	VIYR	VHYR	VQDM	VGLN
9	HPGY	KHVQ	KLTR	QGST	WVYE	WKHI	WTLF	WMYR	WNHV	WHEG
0	MEMY	LELS	VETC	HYRK	SSFI	YIFL	YWPI	YRQI	YWEP	YKVT
	(a)	*(b)*	*(c)*	*(d)*	*(e)*	*(f)*	*(g)*	*(h)*	*(i)*	*(j)*

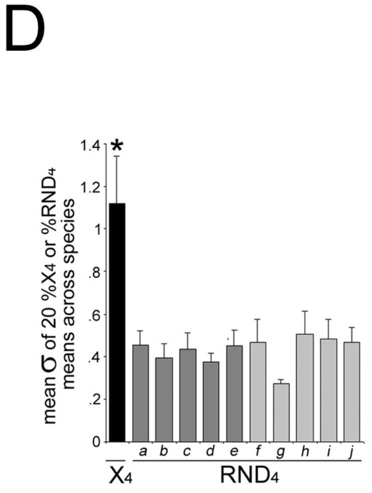

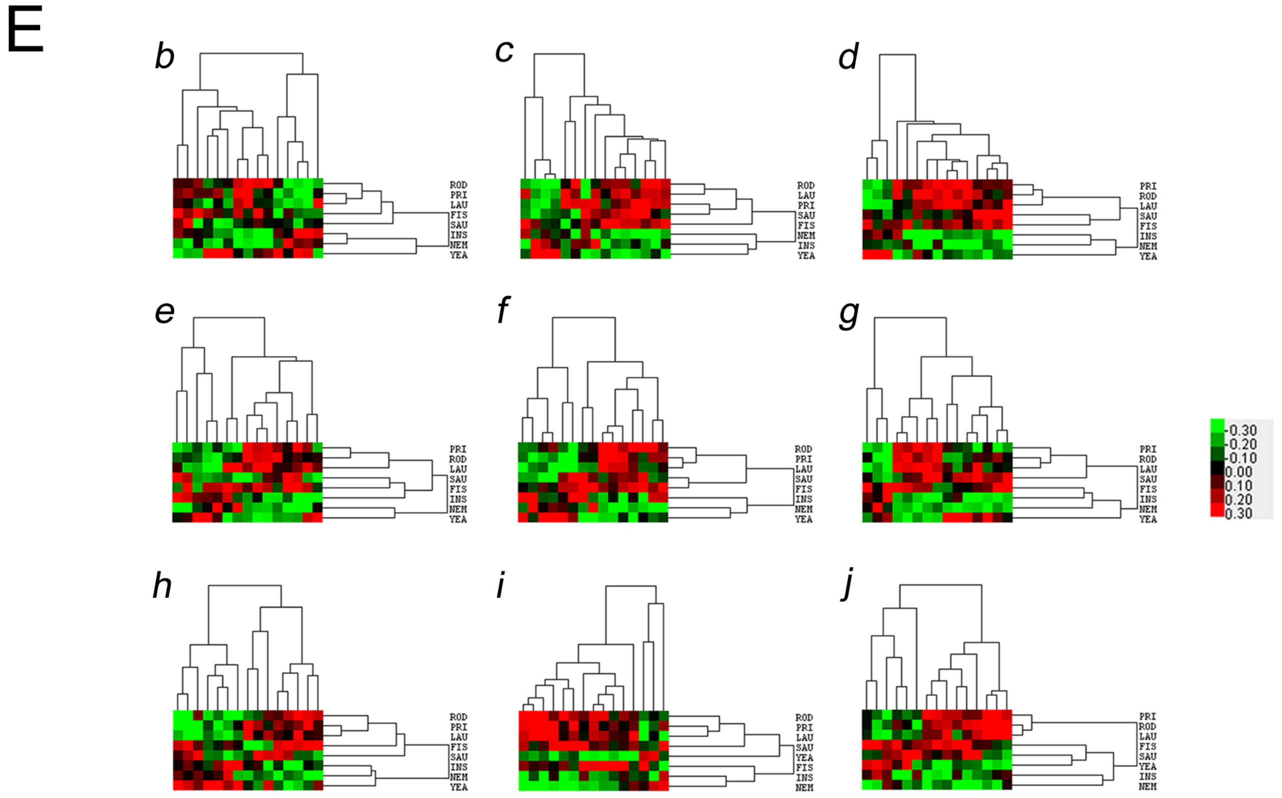

Supplemental figure 8

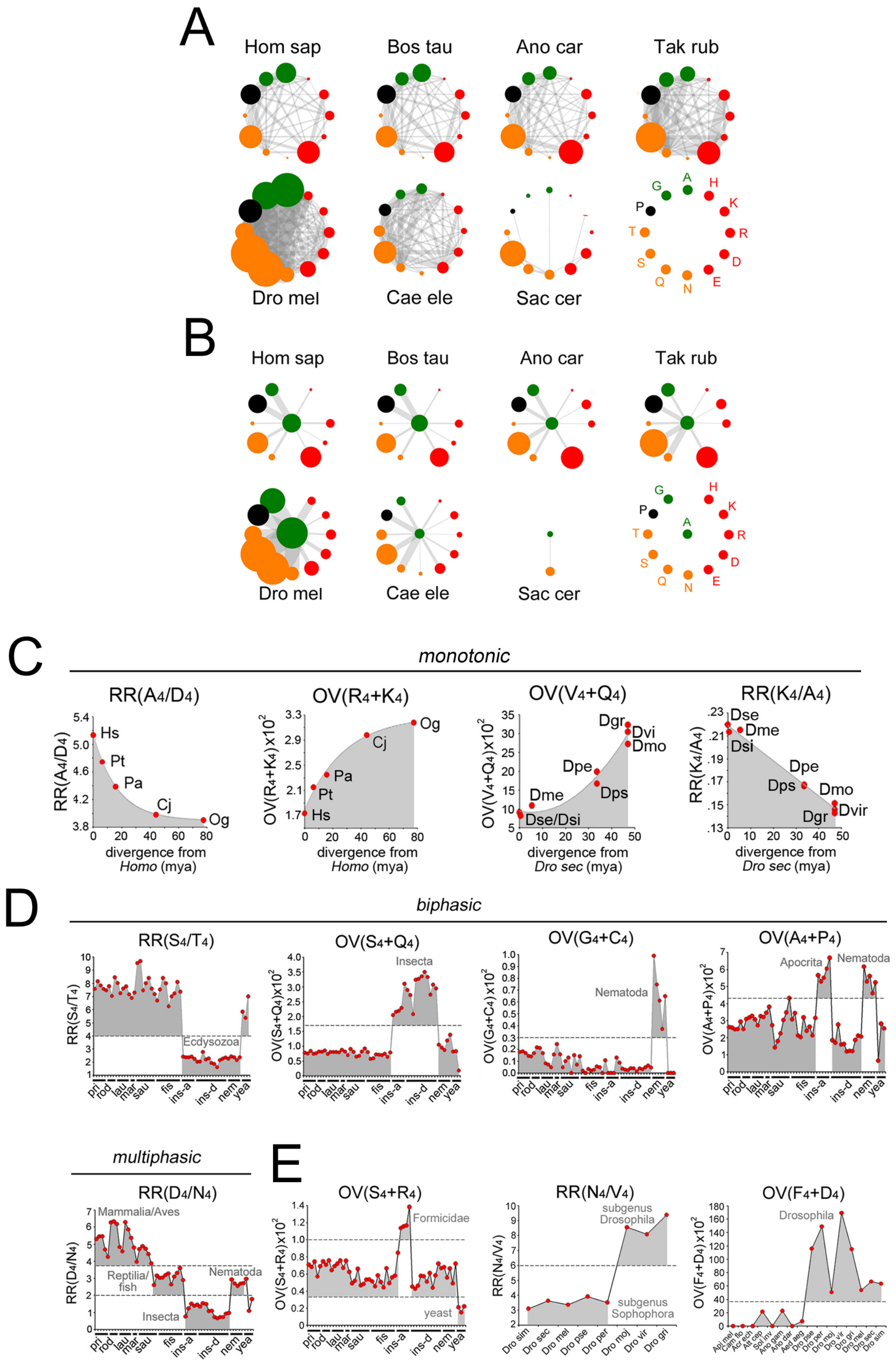

Supplemental figure 9

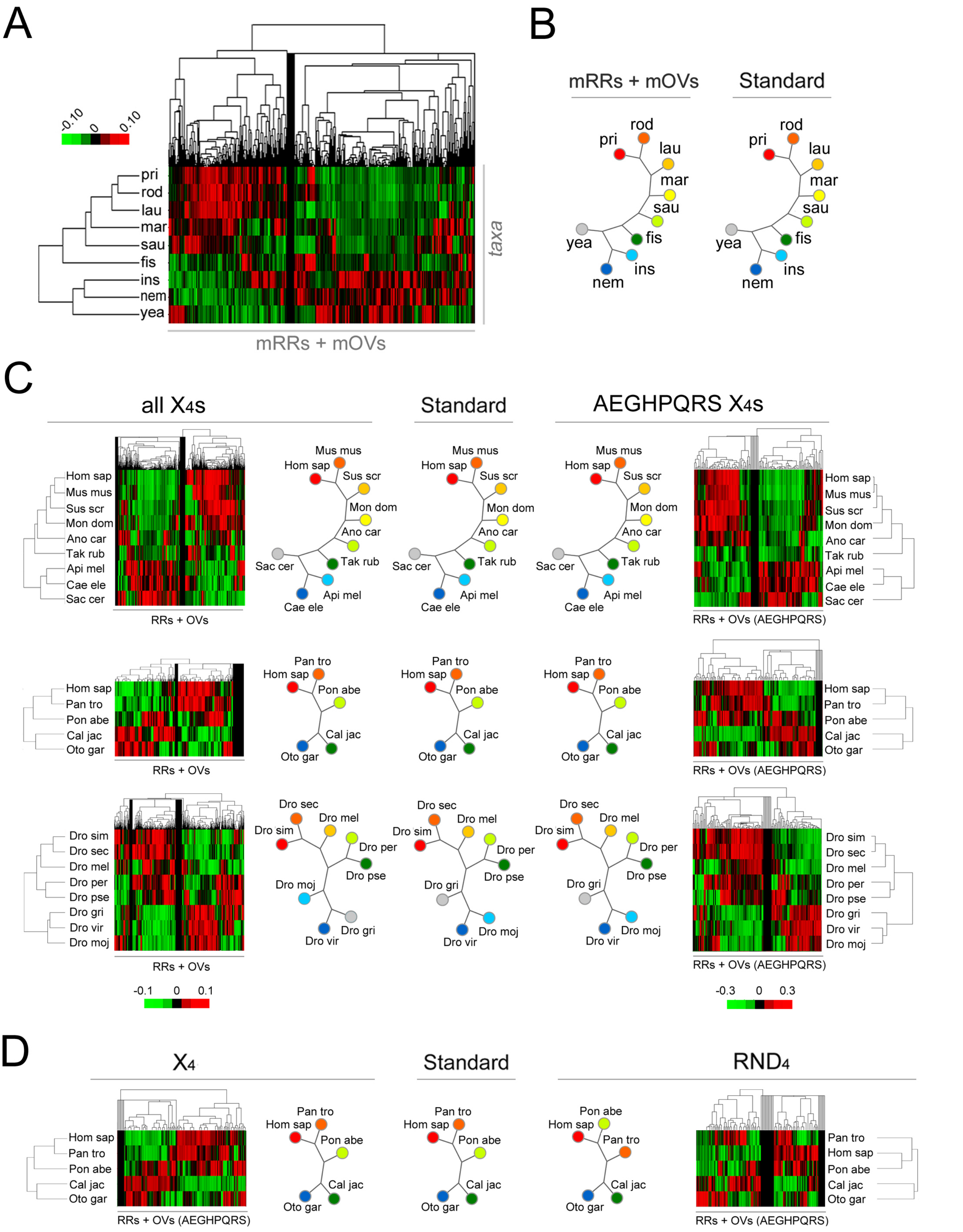

Supplemental figure 10
